# Neutral Ligand Triggered Low-Dimensional Reconstruction for Improving the Efficiency and Stability of Perovskite Solar Cells

**DOI:** 10.1021/acsaem.4c01301

**Published:** 2024-10-18

**Authors:** Ran Wang, Zhenyu Jia, Ben F. Spencer, Dawei Zhao, Andrew G. Thomas, Osama M. Alkhudhari, David J. Lewis, Robert J. Cernik, Ashwaq Alanazi, Brian R. Saunders

**Affiliations:** † Department of Materials, University of Manchester, Engineering Building A, Manchester M1 7HL, U.K.; ‡ Center for Micro-Nano Systems, School of Information Science and Technology (SIST), Fudan University, Shanghai 200433, P. R. China; § Photon Science Institute, the Henry Royce Institute, University of Manchester, Manchester M13 9PL, U.K.; ∥ Department of Chemistry, College of Science, Taif University, Taif 21944, Saudi Arabia

**Keywords:** perovskite solar cells, low-dimensional, imidazole, remodeling, efficiency, stability

## Abstract

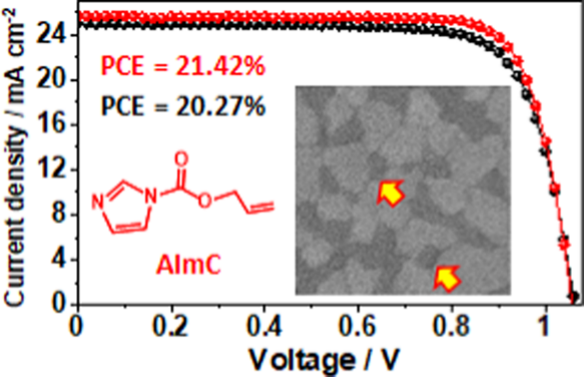

Perovskite solar cells (PSCs) offer a potentially large-scale method for producing low-cost renewable energy. However, stability challenges currently limit their practical application. Consequently, alternative methods for increasing the PSC stability are urgently needed. Compared with three-dimensional (3D) perovskites, low-dimensional (LD) perovskites have been shown to have higher stability. In this study, a LD/3D hybrid perovskite strategy is used that involves post-treating the Cs_0.05_(FA_0.98_MA_0.02_)_0.95_Pb(I_0.98_Br_0.02_)_3_ perovskite with a neutral allyl 1H-imidazole-1-carboxylate (AImC) ligand. We show that this neutral organic spacer molecule has two key roles. AImC acts as a solvent and triggers localized reconstruction to produce a LD capping layer in one postprocessing step. AImC prolongs the carrier lifetime and reduces trap-assisted recombination. As a result, the PSCs containing AImC achieve a maximum power conversion efficiency (PCE) of 21.42% compared to 20.27% for the control device and show significantly decreased hysteresis. AImC also greatly increased the stability of the films and devices to air, moisture, and heat. The results of this study imply that neutral amine liquids that have the correct solvating and ligating properties have good potential to improve the PCE and stability of the PSCs.

## Introduction

Organic–inorganic metal halide perovskite solar cells (PSCs) have been extensively investigated in the past 12 years with the result that their power conversion efficiency (PCE) has increased from 9.7 to 26.7%.^1−3^ This improvement is due to their excellent light absorption, low exciton binding energy, high tolerance to defects, mobile ion management^4^ as well as ease of fabrication,^5^ and improved defect passivation strategies.^6^ However, the stability of three-dimensional (3D) perovskite-based PSCs is still much less than that of silicon solar cells, which represent the gold standard for solar cell stability. A major source of PSC instability is the growth of defects at the grain surfaces during and after annealing.^7^ Such defects not only accelerate perovskite instability to humidity, high temperature, and light but also decrease the PCE.^8^ Because the stability challenge is not yet solved for PSCs, new methods for improving stability continue to be highly desirable. Such methods include PSCs that contain 2D/3D heterostructures which are gaining increasing prominence.^9−13^ Here, we investigate the effects of a new neutral imidazole ligand strategy on the properties and performance of low-dimensional (LD)/3D perovskite films and devices.

Generally, LD perovskites have a greater formation energy (and intrinsic stability), increased hydrophobicity and decreased ion migration compared to 3D perovskites.^14^ The most common method for constructing 2D perovskites uses spacer cations from ammonium salts^15^ such as phenylethylammonium iodide, octylammonium iodide, and butylammonium iodide.^16−18^ The quasi-2D Ruddlesden–Popper (RP) type is the most studied 2D perovskite and has the general structural formula of (*A*′)_2_(*A*)_n–1_Pb_*n*_*X*_3*n*+1_, where *A*′ represents a long chain organic cation.^19,20^ The parameter, *n*, is the number of octahedral inorganic sheet layers separated by the organic spacers;^5,9,21,22^*A* denotes the small cations (e.g., CH_3_NH_3_^+^, CH(NH_2_)_2_^+^, or Cs^+^) and *X* represents a halide.^23^ Pure 2D perovskites do not provide very high PCEs because the organic spacers inhibit out-of-plane charge transport due to dielectric confinement via multiple quantum well structures.^18^ This limitation led to the development of 2D/3D perovskites with a 2D capping layer^9,24^ that increased perovskite stability and PCE. While there have been many reports concerning the preparation of LD capping layers using organic charged ammonium spacer cations,^9,24^ there are far fewer studies involving neutral ligands.

**Figure 1:**
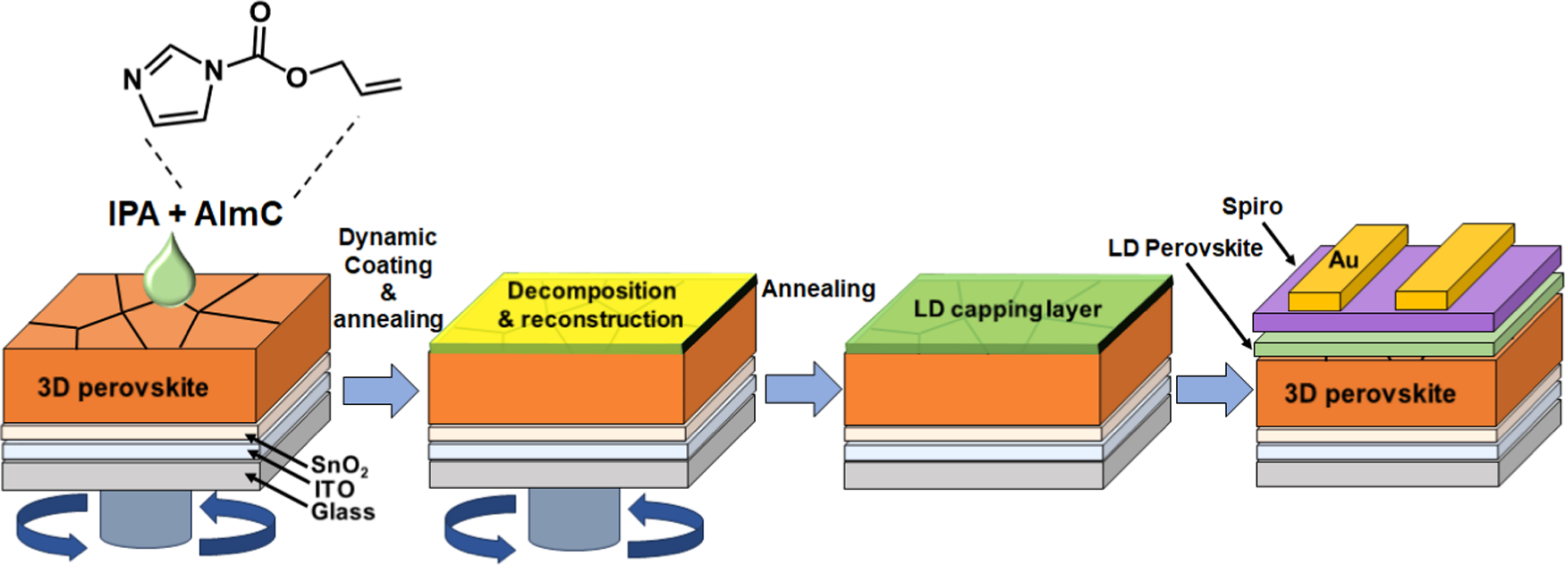
Schematic of the 3D perovskite film post-treated with allyl 1H-imidazole-1-carboxylate (AImC) to form a mixed LD/3D perovskite surface via a surface reconstruction process followed by *n-i-p* PSC construction. IPA is isopropanol.

The neutral organic spacer *n*-butylamine has been used to prepare 2D/3D perovskites.^25^ Huang et al. noted that an advantage of using neutral ligands is that, unlike ionic ligands, they avoid introducing excess iodine.^25^ Imidazole has an aromatic structure and is highly polar, containing a basic nitrogen^26^ that is suitable for coordination with species such as Pb^2+^. Cationic imidazole spacer ligands have been previously studied in 2D/3D perovskites.^27,28^ Wang et al. used an imidazole-based diiodide to prepare efficient inverted PSCs. Interestingly, they reported that the neutral form of the imidazole completely decomposed the perovskite^29^ and was not suitable for use. In an earlier study, Zhou et al. demonstrated that perovskite surfaces could be rendered ultrasmooth by methylamine (MA)-triggered perovskite decomposition and reconstruction.^30^ Inspired by these reports, we hypothesized that a neutral imidazole with a less strong binding ability than the neutral *N*-(3-aminopropyl)-imidazole used by Wang et al.^29^ would enable reconstruction of an LD capping layer. We investigate this hypothesis in the present study using the neutral imidazole ligand allyl 1H-imidazole-1-carboxylate (AImC, Figure 1).

Here, we investigate the ability of AImC to provide a spacer-assisted perovskite localized LD capping layer reconstruction in one postprocessing step. We show that AImC ligand improves the smoothness of the perovskite surface and provides a LD perovskite capping layer with tunable thickness. The LD layer passivates the perovskite film, adjusts the band gap, and enhances the stability of the perovskite. Steady-state photoluminescence spectroscopy (PL) and time-resolved photoluminescence (TRPL) results show that defect densities are decreased, and carrier extraction rates are improved by the capping layer. These benefits contribute to the PCE increasing from a control value of 20.27 to 21.42% when AImC is included. The storage stability of unencapsulated devices containing AImC have a projected time for reaching 80% of the original value (*t*_80_) of more than six months. We show that AImC acts as a dual-role spacer ligand that combines the advantages of smooth surface reconstruction previously reported for MA^30^ and postprocessing morphological reconstruction associated with LD capping layer formation. It follows from the results presented in this study that neutral amine liquids that have an intermediate perovskite solvating ability and good ligating capability (such as AImC) have excellent potential to improve the PCE and stability of PSCs.

## Experimental Section

### Materials

Tin(IV) oxide (SnO_2_, 15% in H_2_O colloidal dispersion) was obtained from Alfa Aesar. Formamidinium iodide (FAI, 99.5%), methylammonium bromide (MABr, 99.5%), and indium tin oxide (ITO, 20 × 15 × 1.1 mm^3^) substrates were purchased from Ossila. Allyl 1H-imidazole-1-carboxylate (AlmC, 95%), 2-propanol (IPA, anhydrous, 99.5%), cesium iodide (CsI, 99.999%), methylammonium chloride (MACl, 99.5%), lead bromide (PbBr_2_, 99.999%), spiro-MeOTAD (99%), 4-tertbutylpyridine (TBP, 96%), bis(trifluoromethane)sulfonimide lithium salt (Li-TFSI, 99.95%), FK 209 Co(III) TFSI, 98%), dimethylformamide (DMF, 99.8%), dimethyl sulfoxide (DMSO, 99.9%), isopropanol (IPA, 99.5%), and chlorobenzene (CBZ, 99.8%) were all purchased from Sigma-Aldrich. Lead iodide (PbI_2_, 99.99%) was purchased from the Tokyo Chemical Industry. All materials were used as received. Water was ultrahigh purity and deionized.

### LD/3D Perovskite Films Preparation

The ITO glass substrate (15 Ω·sq^–1^) was cleaned in Hellmanex aqueous solution (2%), acetone, ethanol, and IPA by ultrasonic cleaning for 15 min each, then dried with nitrogen stream and treated with UV-ozone for 20 min. SnO_2_ solution (100 μL, 2.67 wt %) was spin-coated on UV-ozone-treated ITO substrate at 3000 rpm and then annealed at 150 °C for 30 min. The annealed substrates were immediately transferred to the glovebox after being retreated with UV-ozone for 15 min. We prepared triple-cation perovskite films (Cs_0.05_(FA_0.98_MA_0.02_)_0.95_Pb(I_0.98_Br_0.02_)_3_). First, CsI (0.0195 g, 0.075 mmol), PbI_2_ (0.68 g, 1.48 mmol), FAI (0.24 g, 1.40 mmol), MABr (0.0032 g, 0.029 mmol), and PbBr_2_ (0.011 g, 0.030 mmol) were dissolved in a mixture of 1.0 mL of DMF/DMSO (*v/v* 4:1), and 5.0 mol % excess PbI_2_ and 15.5 mol % MACl were added to improve the crystallization quality. The vial was transferred to a nitrogen glovebox (humidity ∼10.0%), stirred, and heated at 55 °C for 2 h, and then a 0.22 μm PTFE filter was used to filter the perovskite precursor solution. A one-step deposition method was used with a N_2_-filled glovebox with a low humidity (∼4 RH%, 20–25 °C). The precursor was spin-coated on the ITO/SnO_2_ substrate at a speed of 1000 rpm, 10 s with 200 rpm/s acceleration and 6000 rpm, 20 s with 1000 rpm/s acceleration, and CBZ (200 μL) was dropped slowly onto the films 10 s before the end of the spin-coating. The films were annealed for 40 min at 120 °C. The same procedure was used to prepare thinner 3D perovskite films using precursor solution with a total concentration of 0.50 M. Unless otherwise stated, this work focuses on the thicker 3D perovskite films. AImC solutions with different concentrations of AImC were prepared in IPA in vials and stirred at room temperature for 5 min. Then, 70 μL of the neutral spacer solution was dropped quickly on the annealed perovskite film with dynamic spin-coating at 5000 rpm for 30 s, followed by thermal annealing at 100 °C for 6 min to form the LD perovskite. The formation of the LD layer was also conducted in a N_2_-filled glovebox.

### Perovskite Device Preparation

We used zinc powder and HCl to etch the ITO substrates. The steps for preparing the perovskite films were described above. The hole transport layer (HTL) was deposited on top of the light-absorbing layer of the perovskite. Accordingly, Spiro-OMeTAD (85 mg) was added to 1.0 mL of CBZ in a glovebox and mixed with tBP (34 μL), Li-TFSI (22 μL), and FK 209 Co (III) TFSI (18 μL) at 45 °C for 5 min. Filtering (PTFE, 0.22 μm) was used before solution use. The Spiro solution (75 μL) was dynamically spin-coated on the perovskite film at 3000 rpm for 30 s (2000 rpm/s acceleration). The Spiro layer was oxidized overnight in a dark box, and phosphorus pentoxide (P_2_O_5_) was placed in the box to remove excess water. Finally, an 80 nm layer of Au was thermally evaporated at a rate of 5.0 Å·s^–1^ on the HTL to form the back electrode. The area of the device was 0.079 cm^2^, as defined by a metal shadow mask.

### Characterization

X-ray photoelectron spectroscopy (XPS) was conducted on an ESCA2SR spectrometer (Scienta Omicron GmbH) using monochromatic Al Kα radiation (1486.6 eV, 25 mA emission at 300 W, 1 mm spot size) at a base vacuum pressure of ∼1 × 10^–9^ mbar. Atomic force microscopy (AFM) was performed on Multimode 8, using aTap300Al-G-10 probe. Kelvin probe force microscopy (KPFM) was performed with Multimode 8 with frequency modulated KPFM (peak force tapping mode). An Au sample was used as a reference sample for calibration (5.22 eV). Scanning electron microscope (SEM) images were obtained with a Tescan Mira 3 SC. Backscattered electron SEM (BSE) SEM images were obtained with an Ultra 55 Carl Zeiss Sigma FEG-SEM. X-ray diffraction (XRD) data were acquired with a PANaytical X′Pert Pro X-ray diffractometer. The sample for mixing AImC with PbI_2_ for XRD was prepared by dissolving AImC (40 mg) and PbI_2_ (40 mg) in IPA, with stirring at 55 °C for 2 h. Then, 0.2 mL of the mixture was dropped onto a glass substrate and heated at 120 °C for 1 h. Fourier transform infrared (FTIR) transmission spectra were measured by using a Bruker α FTIR spectrometer. The mixed AImC/PbI_2_ sample was prepared by dissolving AImC (40 mg) and PbI_2_ (20 mg) in DMF (1.0 mL). Then, the mixture solution was stirred at 35 °C. After 1 h, it was dried on a clean glass at 100 °C for a further 1 h. AImC liquid was dropped on the clean glass directly to dry for 1 h at 100 °C. UV–visible spectra were measured by using an Agilent Cary 60 UV–visible spectrophotometer. Steady-state photoluminescence spectroscopy (PL) was measured using an Edinburgh Instrument FLS980. The excitation wavelength was 470 nm. Time-resolved photoluminescence (TRPL) data were also measured using the FLS980 and an excitation wavelength of 405 nm. All UV–visible, PL, and TRPL spectroscopy measurements were recorded using glass/ITO/SnO_2_ substrates. For all PL measurements, the light was incident on the film side. Contact angle measurements were performed with a drop of water (60 μL) on the film surface by using a Kruss DSA100.

### Device Measurements

*J*-*V* curves were generated by a Keithley 2420 source meter while simulating AM 1.5G sunlight (100 mW·cm^–2^) using an Abet solar simulator. The instrument was calibrated and corrected for spectral irradiance mismatch by using a certified Oriel Si reference cell. The device area is 0.079 cm^2^. *J–V* data and metrics are shown from the reverse scan, unless otherwise stated. For all stability tests, three devices were used per system. A Keithley 2420 source meter was also used for measuring the light dependence for devices. A Newport QuantX-300 instrument was used for external quantum efficiency (EQE) measurements. All devices were measured in ambient air without encapsulation.

## Results and Discussion

### Characterization of the LD Capping Layer

**Figure 2:**
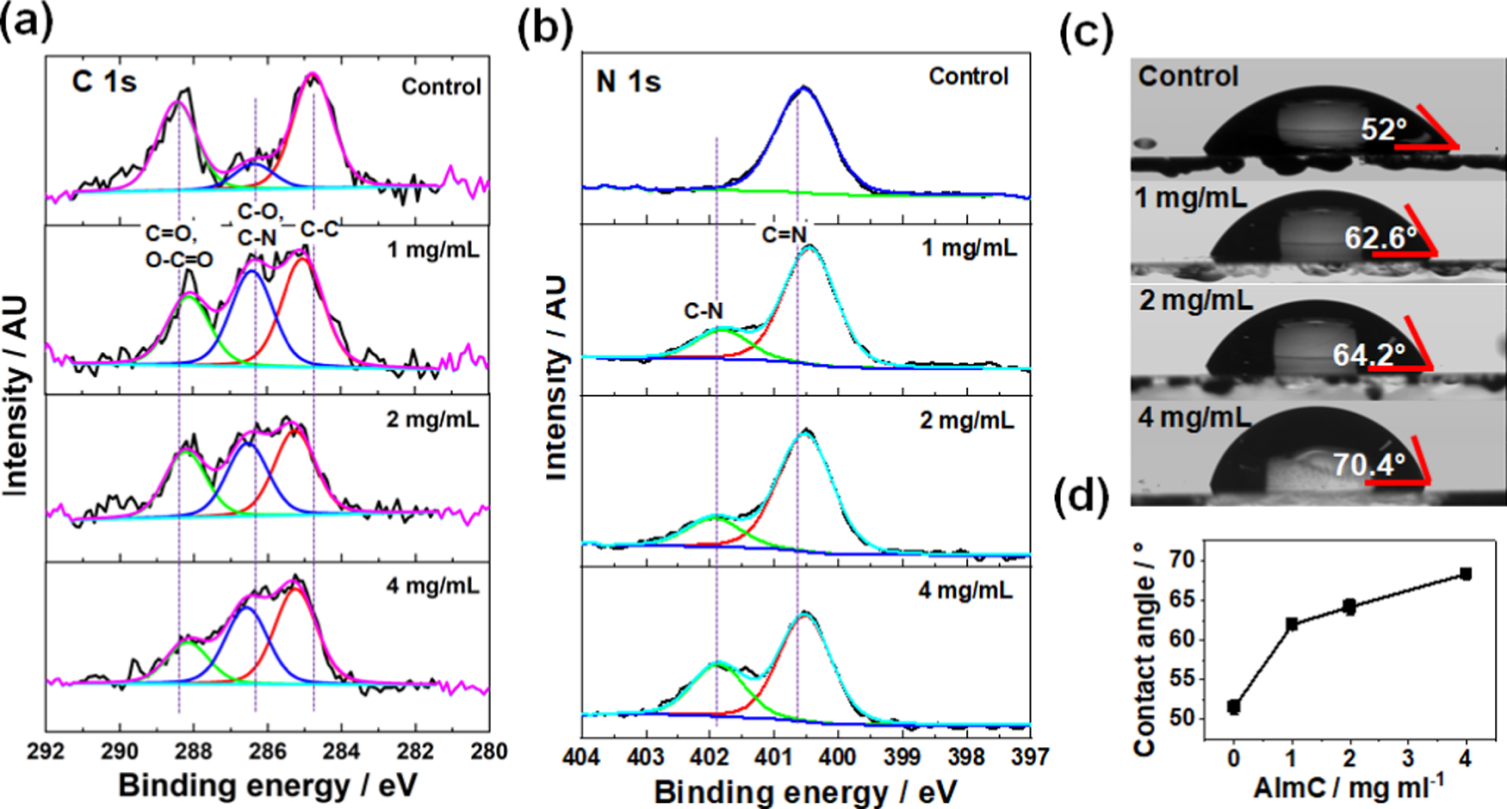
XPS spectra of (a) C 1s and (b) N 1s for the control perovskite film and AImC-treated LD/3D perovskite films. The concentrations are shown. Black lines represent the measured curve, and colored lines represent the fitted lines. (c) Photographs of water droplets on the perovskite surfaces. (d) Contact angles from part (c).

Perovskite films were prepared using post-treatment using IPA solutions containing 1, 2, and 4 mg/mL AImC as depicted in Figure 1. We first used C 1s and N 1s XPS data to establish the presence of AImC at the perovskite surface. In the C 1s spectrum (Figure 2a), a strong C–C peak (284.8 eV) can be observed for the control perovskite.^31^ The change of C–C peak position (from 284.8 to 285.3 eV) as the AImC concentration increases shows that the chemical environment on the perovskite surface changed.^32^ The C=O peak (288.5 eV) for the control perovskite sample is ascribed to adventitious carbon,^33^ and the 286.4 eV peak is due to the C–N of formamidinium (FA).^34,35^ With increasing AImC concentration, the intensity of C–N peak increases, indicating that this C–N species is from the imidazole ring of AImC. After AImC treatment, the C=O peak shifts from 288.5 to 288.2 eV. The latter peak is attributed to the C=O group from AImC.^36^ The N 1s spectra (Figure 2b) show a peak at 400.6 eV of C=N from FA. A new C–N peak is evident at 401.8 eV, which is attributed to a protonated amine in AImC.^29^ Furthermore, both the C 1s and N 1s spectra confirm that AImC has been successfully introduced to the surface of the perovskite films. It follows that the surface should be more hydrophobic. Accordingly, we measured water contact angles of the perovskite films post-treated with different concentrations of AImC (Figure 2c,d). The water contact angle increases from 52° for the control to 70.4° for the 4 mg/mL system. Hence, the introduction of AImC increased the film hydrophobicity, confirming that AImC was present at the perovskite surface.

**Figure 3:**
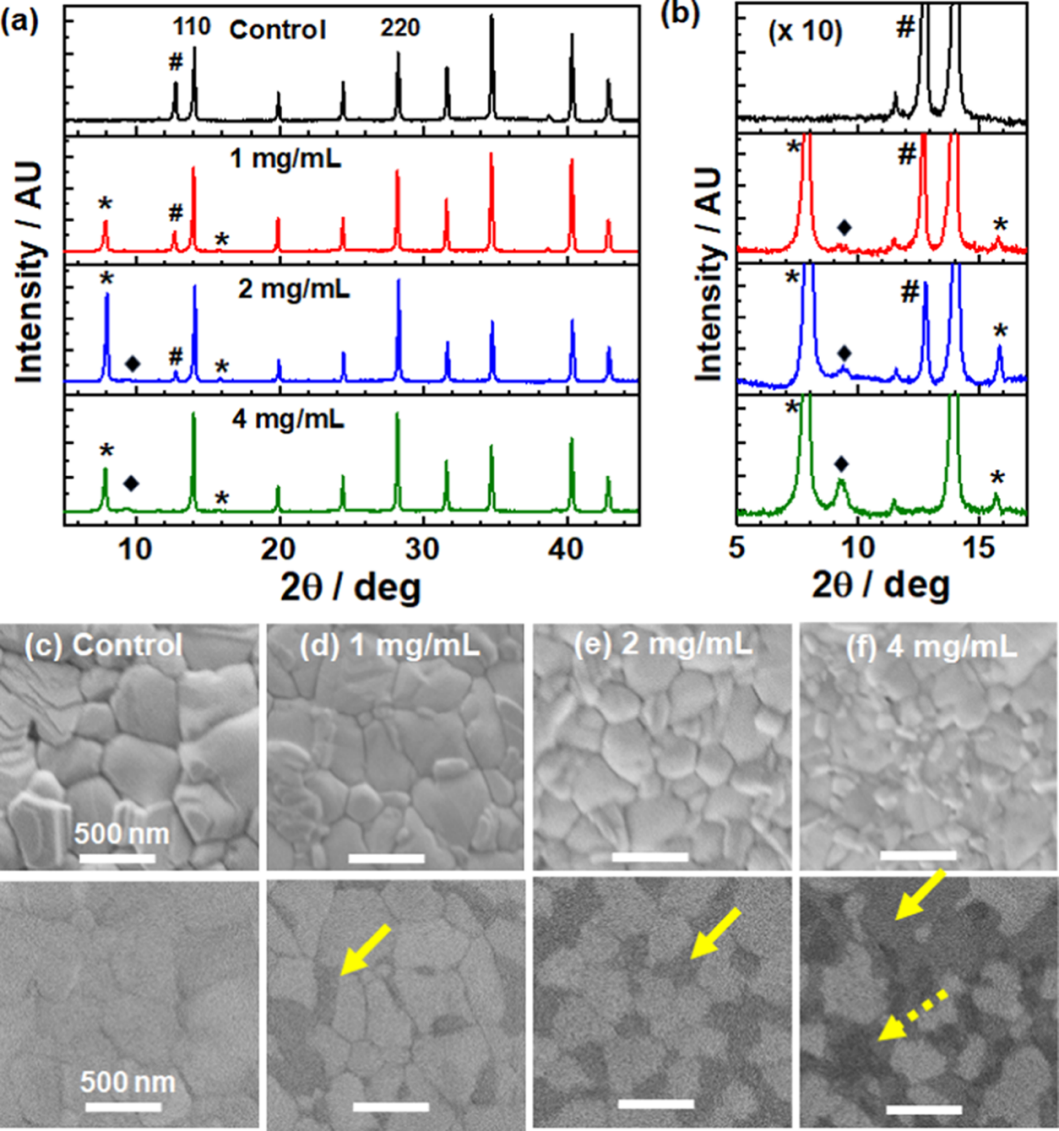
(a) XRD patterns of perovskite films treated with different concentrations of AImC. (b) Magnified XRD patterns at low angles from 5 to 17°. * indicates LD perovskite signal, diamonds indicate interphase (see text). # indicates PbI_2_. (c)–(f) Top-view SEM images (top row) and BSE SEM images (bottom row) of the perovskite films. Yellow arrows highlight the LD phase, and the yellow-dotted arrow highlights a different LD phase.

XRD patterns of perovskite films are shown in Figure 3a,b. For all films, the perovskite peak (110) is observed at 14.1°. There is a small-angle diffraction peak at 7.9°, which is assigned to LD perovskite.^37^ Indeed, the 7.9 and 15.9° fingerprint reflections are tentatively identified as 2D perovskite. Our justification for this proposal is provided in Additional Note 1 of the Supporting Information. The signal at 12.7° is due to PbI_2_. Interestingly, the PbI_2_ signal intensity decreased as the AImC concentration increased, which shows that PbI_2_ was consumed as the LD phase was produced.

Top-view SEM was used to observe the morphologies of perovskite films with and without AImC treatment (Figure 3c–f, top row). When post-treated with 1 mg/mL AImC, fine strip-shaped crystals grew from the surface and grain boundaries of the 3D perovskite. At concentrations of 2 and 4 mg/mL, the quantity of smaller crystals increased. Backscattered electron (BSE) SEM images (Figure 3c–f, bottom row) were measured to clearly distinguish between the phases.^38^ BSEs are re-emergent beam electrons that are scattered back into the same hemisphere that contained the incident electron beam.^39^ BSEs respond to composition, with the proportion of such electrons increasing with atomic number. Hence, high atomic number sample regions appear bright, whereas low atomic mass regions appear dark. The average atomic number per unit volume will decrease as the perovskite changes from 3D to lower dimensionality, and this is the basis for using BSE SEM to qualitatively assess composition. Darker crystals and grains are evident in Figure 3c–f (yellow arrows) for the AImC-containing systems, which are due to LD perovskite. It is likely that the lighter regions (3D perovskites) are covered by a thin layer of LD perovskite. For the 4 mg/mL film, new crystals of different brightnesses appeared in addition to the LD phase (yellow-dotted arrow), which is a different phase and is referred to here as LD interphase. Tapping mode AFM was used to examine the effect of AImC treatment at different concentrations on the film surface morphology (Figure S1). The control perovskite film shows a rougher surface with a root-mean-square (RMS) roughness of 27.9 nm. Importantly, the roughness decreased to 24.6 and 19.9 nm with the post-treatments of 1 and 4 mg/mL AImC, respectively, showing that AImC improved the smoothness of the perovskite films. SEM cross section images were obtained (Figure S2). Full grains that extended across the whole film are evident. The film thickness (Figure S2(e)) was a maximum for 1 mg/mL AImC (512 nm).

### Neutral Ligand Triggered Reconstruction Mechanism for LD Capping Layer Formation

We investigated the ability of AImC to react with the 3D perovskite. Because AImC was dissolved in IPA for post-treatment (Figure 1), we measured the evaporation rates of both IPA and AImC (which is a liquid) during heating at 100 °C. IPA fully evaporated in 255 s under the conditions of the experiment (Figure S3(a)). In contrast, the relative mass ratio of AImC changed slowly and 53% of the initial mass remained after 25,200 s (7 h). It follows that during post-treatment of the 3D perovskite, the IPA evaporates rapidly, and most of the initial liquid AImC that was added is retained on the surface and is available for LD perovskite reconstruction. Hence, *liquid AImC will cover the perovskite surface after the IPA evaporates*.

It is noted that the N–C bond in imidazolides is weak.^40^ We investigated whether significant decomposition of AImC occurred during heating at 100 °C for 25,200 s by measuring FTIR spectra at selected time intervals (Figure S3(b)). There was no significant change in the spectra in the first 300 s. This indicates that little, if any, decomposition of AImC occurred during the annealing of the AImC-treated perovskite films. Further, the bands due to C=O and CH=C were unaffected by heating over the entire 25,200 s period. However, the bands at 1595 and 1080 cm^–1^ became less strong, although they were still present. Hence, some decomposition of AImC may have occurred when heated at 100 °C for an extended time (e.g., 7 h).

We also investigated whether AImC can react with the perovskite. One drop of IPA (100%), AImC/IPA solution (4 mg/mL), and pure AImC liquid (100%) was added to 3D perovskite films and photographs recorded as a function of time (Figure S3(c)). IPA wetted the perovskite surface and did not induce a noticeable change. For the 4 mg/mL AImC/IPA solution, a small fraction of the wetted perovskite became brown after 30 s. Crucially, pure AImC liquid caused yellowing of the perovskite and partially reacted with the film surface within 30 s. This experiment shows that *AImC partially reconstructed the 3D perovskite*. We contend that AImC behaves broadly similarly to gaseous CH_3_NH_2_^30^ in terms of perovskite surface reconstruction. Accordingly, the relative smoothness improvement noted from the AFM data by AImC post-treatment (Figure S1) is due to reconstruction of the LD perovskite.

It follows that AImC triggers a remodeling of the surface structure and reconstruction of an LD capping layer. We tested this assumption by adding AImC to 3D perovskite powder that had been scrapped off the substrate and redispersed in IPA. Photographs of the dispersions and UV–visible spectra were recorded after the AImC additions (Figure S4(a)). The dispersion changed from dark black to a mustard color as the AImC concentration increased. The spectra show the emergence of a lower wavelength maximum at about 360 nm (Figure S4(b)) and the loss of the high wavelength perovskite absorption (Figure S4(c)), which is evidence for a direct reaction between the 3D perovskite and AImC. We note that the absorption at 360 nm is at a higher wavelength than that for PbI_2_ (which occurred at 320 nm, Figure S4(b)) and is likely due to LD perovskite. This proposal is supported by the PL spectrum of the perovskite dispersion treated with 14.7 mg/mL AImC that shows a strong maximum at 394 nm (Figure S4(d)).

Our proposed mechanism for the transformation of 3D perovskite films into LD/3D systems upon the addition of AImC is depicted in Figure S5. AImC adsorbs to the 3D surface and reacts with the surface 3D perovskite film, and the surface structure remodels to LD perovskite.^41^ As the process continues, the Pb–I octahedra are reconstructed.^42^ A benefit afforded by AImC is that its dual role as solvent and reactant at the 3D surface enhances LD reconstruction.^30^ Such an increase in the reactant concentration is expected to increase the nucleation site concentration for the LD phase and is likely a key reason for the decreased surface roughness.

IPA plays an important role in the delivery of AImC to the surface. We note that large amounts of AImC caused excessive reconstruction of the 3D perovskite film, as evidenced by Figures S3(c) and S4. We sought *limited* reconstruction of the uppermost 3D perovskite surface to achieve a limited LD layer. Consequently, restricting the amount of AImC present at the surface was crucial, which required delivery of a thin layer of AImC uniformly distributed over the 3D perovskite surface prior to reaction. To achieve this, IPA was used as a *flow enhancer* and *diluent* for AImC. Enhancing flow during spin-coating requires lowering the viscosity. IPA has a viscosity lower than that of AImC as can be seen from a tube inversion test where the times required for AImC and IPA to travel the distance of an inverted NMR tube are compared (Figure S6(a)). AImC required approximately 100 s to flow to the bottom of the tube, whereas IPA only required 20 s (Figure S6(b)). By dissolving AImC in IPA, the lower viscosity solution gave a more uniform coverage of the 3D perovskite surface by IPA than could be achieved using pure AImC alone. Furthermore, IPA acted as a *diluent* enabling much lower AImC concentrations (1–4 mg/mL) to be used than for pure AImC liquid (1120 mg/mL). Dilution provided good tunability and prevented excessive reconstruction from occurring at the 3D surface. Hence, using IPA enabled limited and optimal LD remodeling of the uppermost 3D perovskite surface to be achieved by AImC. Further comments regarding the reconstruction mechanism are provided in the next section.

### AImC Strongly Interacts with Pb^2+^, Passivates Perovskite, and Decreases Defect Density

**Figure 4:**
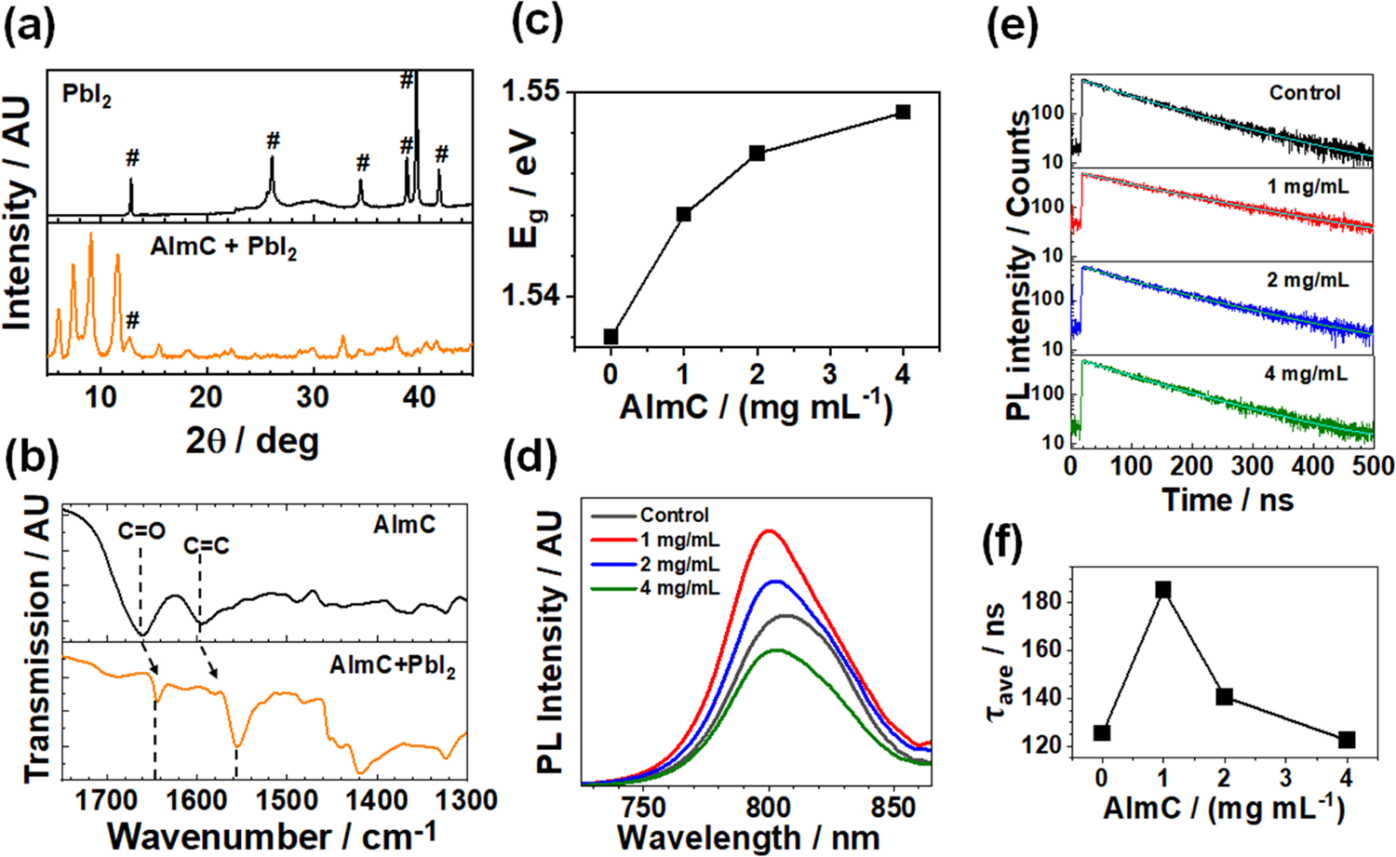
(a) XRD patterns for AImC mixed with PbI_2_. # indicates PbI_2_ peaks. (b) FTIR spectra of AImC and an AImC/PbI_2_ mixture. (c) Band gap values for the systems. (d) PL spectra of the control film and AImC-treated LD/3D films. (e) TRPL data and fits of the control film and AImC-treated LD/3D films. (f) Variation of τ_ave_ with the AImC concentration.

To probe the interaction between AImC and Pb^2+^, we mixed AImC with PbI_2_ and measured the resulting XRD pattern and FTIR spectra. The XRD pattern for the AImC/PbI_2_ mixture (Figure 4a) shows multiple new peaks in the range of 5° to 15°. None of these new peaks match those identified for the LD perovskite in Figure 3b. The former data are strong evidence for complex formation between PbI_2_ and AImC, which likely involves AImC binding to Pb^2+^. We propose that the XRD pattern is the result of the formation of a nonperovskite adduct according to the following reaction.
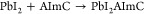
1

FTIR spectra of pure AImC and a mixture of AImC with PbI_2_ were collected to further investigate the interaction between AImC and PbI_2_ (Figures 4b and S7). The band at 1661 cm^–1^ is due to the C=O vibration from AImC,^43,44^ and the band at 1595 cm^–1^ is from the C=C group.^45,46^ After mixing with PbI_2_, the C=O band underwent a major shift to 1644 cm^–1^, which is attributed to the coordination of C=O with Pb^2+^.^47^ Furthermore, the C=C vibration moved to 1555 cm^–1^, implying a direct interaction of the electrons in the π-conjugated molecular orbitals with Pb^2+^. Hence, there was a major interaction of Pb^2+^ with both the imidazole and the C=C bonds of AImC.

We used XPS data from the Pb 4f binding energy region to probe the interaction between Pb and AImC in the capping layer. In the Pb 4f XPS spectrum for the control (Figure S8), the binding energies (BE) of 143.2 and 138.3 eV are assigned to the 4f_5/2_ and 4f_7/2_ core levels of Pb^2+^. After post-treatment with AImC (4 mg/mL), the peak values for Pb 4f_5/2_ and Pb 4f_7/2_ shifted slightly toward higher BE values by 0.1 eV, which may indicate an increased Pb^2+^ electron density due to coordination with AImC.^48−50^

While the exact chemical scheme by which AImC transforms 3D perovskite to LD perovskite is not currently known, we provide general comments here. First, AImC interacts very strongly with the 3D perovskite (even at room temperature) and rapidly transforms it to LD perovskite as evidenced by Figures S3(c) and S4. Second, a strong coordination with Pb^2+^ occurred based on Figure 4a,b. The structure of AImC (Figure 1) has important similarities to those of *N*-methy-2-pyrrolidone and 1,3-dimethyl-3,4,5,6-tetrahydro-2(1H)-pyrimidinone, both which interact strongly with FAPI.^51,52^ These similarities are a C=O group bonded to a tertiary nitrogen and a nitrogen-based heterocycle. The AImC oxygen atoms may form hydrogen bonds with the FA cations, enhancing the structural rearrangement of the 3D perovskite that added to the AImC triggers. Furthermore, the formation of LD perovskite requires a cationic organic spacer, and evidence for such a species is present based on the N 1s XPS data (Figure 2b). We suggest that coordination of AImC to Pb^2+^ and hydrogen bonding with FA were involved in LD phase formation. Indeed, the decrease in the peak intensity for PbI_2_ as the AImC concentration increased (Figure 3a) implicates residual PbI_2_ in the spontaneous LD capping layer reconstruction process. Further study of the mechanism underpinning LD formation triggered by AImC is warranted and will be the subject of future work.

The UV–visible spectra for the perovskite films before and after post-treatment with AImC (Figure S9(a)) show generally similar spectra, although the spectra are blue-shifted for the films containing AImC (Figure S9(b)). This indicates less light harvesting in the red region of the spectrum. The blue shift is quantified using Tauc plots (Figure S10), and a gradual increase in the band gap from 1.538 for the control to 1.549 eV after 4 mg/mL AImC post-treatment is apparent from these data (Figure 4c). This blue shift is ascribed to the LD capping layer. We note that an increased band gap is conducive to obtaining a higher open circuit voltage *V*_oc_.^53^

To explore the effect of the LD/3D structure on the recombination charge dynamics, we performed steady-state photoluminescence spectroscopy measurements (Figure 4d). Compared with the control 3D perovskite film, the LD/3D perovskite film treated with 1 mg/mL AImC exhibited significantly enhanced PL intensity, which indicates a decrease of nonradiative recombination.^54^ However, when the concentration was further increased to 2 and 4 mg/mL, the emission intensity decreased gradually (Figure S11).

It is well-known that LD capping layers can give rise to low wavelength UV–visible maxima and PL peaks. We tested for these occurrences using the perovskite films discussed above, as well as thinner films prepared using 0.50 M 3D precursor solution. The latter control and 4 mg/mL films were semitransparent (Figure S12(a)), and a small peak at 370 nm was present for the 4 mg/mL system in the UV–visible spectrum (Figure S12(b)). However, there were no unique peaks in the PL spectra for the thick or thin films evident in the 380–825 nm range (Figure S12(c) and (d)) that could be assigned to LD perovskite. This is attributed to the relatively low amount of LD perovskite on the surface of the films, as shown by the SEM images (Figure 3d–f). Other workers have reported that such peaks may not be observed under similar circumstances.^55^ We stress that the direct reaction of dispersed perovskite powder with high AImC concentration results in spectral changes in low wavelength UV–visible and PL spectral peaks that are consistent with LD perovskite generation as discussed above (Figure S4).

Because PL spectroscopic signatures for the LD capping layer on the 1–4 mg/mL films were not obtainable, we turned to XRD and BSE SEM using a higher AImC concentration of 7 mg/mL to confirm the presence of a LD capping layer (see Figure S13). The XRD data once again show peaks at 7.9 and 15.9° due to the LD phase identified in Figure 3a,b. Furthermore, the SEM and BSE SEM images also show the same features (small grains and lower brightness) that are signatures of the LD phase identified in Figure 3c–f. Hence, for the present systems, it is XRD and BSE SEM data that best reveal the LD capping layer.

The trends shown in Figure 4d are supported by the time-resolved PL (TRPL) data (Figure 4e) which were fitted using the equation^56^
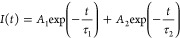
2where *A*_*i*_ is the fractional amplitude for each exponential term. τ_1_ is the bimolecular recombination, and τ_2_ reflects the charge carrier lifetime.^57^ The average carrier lifetime (τ_ave_) is calculated using^58^
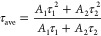
3The decay lifetimes of the TRPL data are plotted in Figure 4f and listed in Table S1. The 1 mg/mL AImC-treated LD/3D perovskite film exhibited a longer PL lifetime (180 ns) than the control film (121 ns), confirming suppressed trap-assisted nonradiative recombination.^59^ When the concentration increased to 2 and 4 mg/mL, the τ_ave_ decreased (Figure 4f), which is due to increased traps caused by thicker LD layers.^60,61^ Hence, the LD capping layers formed by AImC can most effectively passivate the perovskite films when the concentration is 1 mg/mL. We show below that this concentration corresponds to the highest PCE of this study.

### Effect of AImC on Device Efficiency and Defect Concentration

**Figure 5:**
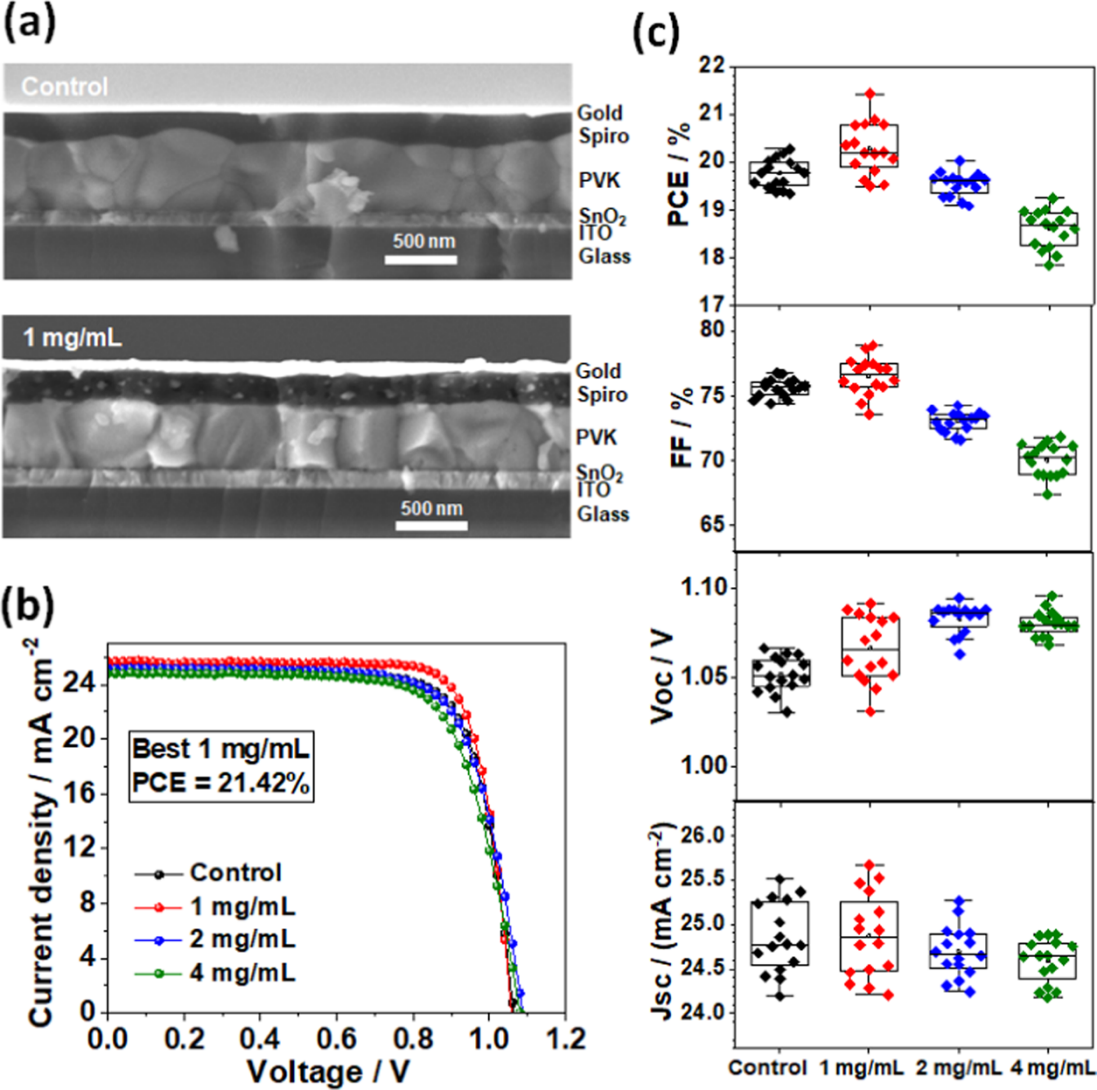
(a) SEM cross-sectional images of the control (top) and AImC (1 mg/mL) perovskite devices (lower). (b) *J*–*V* curves of reverse scan for the highest PCE PSCs investigated. (c) Box plot diagrams of the photovoltaic parameters for all of the devices.

We constructed PSCs using the architecture depicted in Figure 1. Cross-sectional SEM images of the control and 1 mg/mL AImC post-treatment devices (Figure 5a) show that the formation of the LD perovskite layer using 1 mg/mL AImC resulted in a less undulating top perovskite surface compared to the control device, which is in agreement with the AFM data (Figure S1). *J–V* curves are measured under AM 1.5G light and are shown for the champion PSCs (Figure 5b) treated with different AImC concentrations. The control had the highest PCE of 20.27%. For the AImC-treated devices, the maximum PCE was 21.42% achieved for 1 mg/mL (see also Table S2). The average FF values of the cells constructed using 2 and 4 mg/mL of AImC are significantly lower than those of control devices, which may be due to the insulating organic groups that decreased the conductivity of the LD layer.^62,63^ The *V*_oc_ values are highest for the 2 and 4 mg/mL systems (Figure 5c). The *J*_sc_ values decrease slightly with increasing AImC due to relatively obstructed charge transport across the LD layers. While the performance of the 1 mg/mL system is improved by the relatively high *V*_oc_ (Figure 5c) compared to the control, the main factor controlling the PCEs of these devices is the FF. This is demonstrated by the linear relationship between PCE and FF (Figure S14). The integrated *J*_sc_. calculated from the external quantum efficiency (EQE) (Figure S15) are in good agreement with the results obtained from the *J–V* measurements.

We investigated reasons for the 1 mg/mL system providing the best PCE using a suite of different measurements. To measure the surface contact potential difference, Kelvin probe force microscopy (KPFM) was used (Figure S16). The surface scans (Figure S16(a) and (b)) show that the 1 mg/mL system has a more uniform potential (RMS of 0.027 V) across the surface than the control (RMS of 0.056 V), which indicates that the LD layer decreases the magnitude of topological energy level fluctuations. Figure S16(c) shows the potential distribution of AImC-treated and nontreated surfaces. The surface potential increased by 0.090 V from 4.25 V for the control to 4.34 V for the 1 mg/mL film. This indicates that the perovskite film treated with 1 mg/mL AImC has a larger work function difference,^64^ which may optimize band alignment with the HTL^31^ for hole transport, as the Fermi level moves closer to the valence band maximum (*E*_vbm_) of Spiro (see Figure S16(d)). Chen et al.^31^ reported a deeper Fermi level for their 2D-3D perovskite and proposed that under light illumination, the downward shifted hole quasi-Fermi level better matches the *E*_vbm_ of Spiro under illumination, resulting in decreased nonradiative recombination loss due to optimized energy band alignment. The PL data reported here (Figure 4d,f) indicate less nonradiative recombination for the 1 mg/mL system compared to the control.

We measured Suns-*V*_oc_ and *J*_sc_ data (Figure S17) to better understand the effects of trap-assisted recombination.^65^ The relationship between *V*_oc_ and light intensity follows the equation^65^
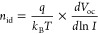
4where *n*_id_ is the ideality factor, *k*_B_ is the Boltzmann constant, *T* is the temperature, *q* is the elementary charge constant, and *I* is the normalized light intensity. When *n*_id_ = 1, electrons and holes show bimolecular recombination. When *n*_id_ > 1, electrons and holes exhibit Shockley-Read-Hall (SRH) recombination.^66−68^ The gradient for the treated AlmC system was determined to be 1.42 *k*_B_*T*/*q* (Figure S17(a)), which is lower than the value of 1.64 *k*_B_*T*/*q* for the control (Figure S17(b)). This demonstrates that trap-assisted recombination loss is significantly reduced by depositing the LD capping layer. The light-*J*_*sc*_ measurements were used to probe the recombination. The *J*_sc_-dependent light intensity is calculated using^69^ the power-law relationship *J*_sc_ ∼ *I*^α^. The α values for the control and LD/3D devices are 0.95 and 0.96, respectively (Figure S17(b)), indicating that bimolecular recombination was decreased due to the passivation effect of the LD layer.^70^

We also constructed electron-only devices for the control and the 1 mg/mL system and measured space-charge-limiting current data. The number density of defects is calculated using^71^
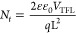
5where ε and ε_0_ are the relative dielectric constant of perovskite (which is^72^ 46.9) and the vacuum dielectric constant, respectively, and *L* is the perovskite film thickness. The latter was taken from the data shown in Figure S2(e). The values for the trap-filled voltage (*V*_TFL_) are taken from Figure S18. The calculated *N*_*t*_ values for the control and 1 mg/mL system are 1.38 × 10^16^ and 5.74 × 10^15^ cm^–3^, respectively, further showing that the LD capping layer decreases the trap density. Hence, the studies above show that the increased PCE for the 1 mg/mL device is due in large part to defect passivation by the LD capping layer, which increased both FF and *V*_oc_. Hence, if the AImC concentration is not too high (e.g., 1 mg/mL in IPA), then the remodeling process discussed above is optimized such that the defect concentration is decreased and charge transport remains sufficiently facile through the capping layer to enable improved PCEs.

### AImC Improves the Stability of PSCs

**Figure 6:**
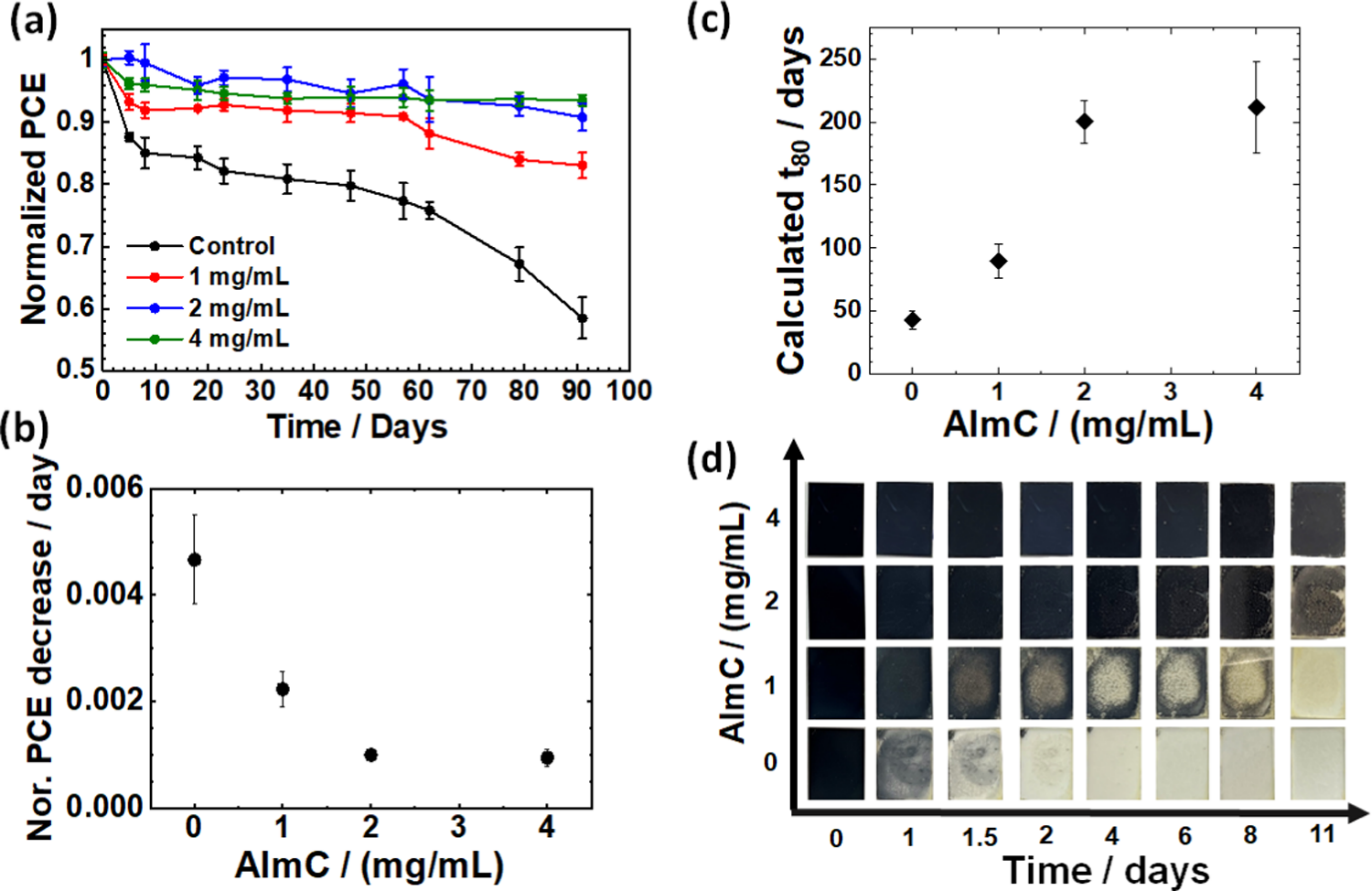
(a) Stability of the PSCs. The unencapsulated devices were stored in the dark and measured at room temperature (40 ± 5% RH). (b) Average PCE decrease rate from part (a). (c) Calculated *t*_80_ values from the data in parts (a) and (b). The *t*_80_ value for the control was obtained by interpolation of the data in part (a). (d) Moisture stability of perovskite films with and without AImC treatment. The films were stored at room temperature and 90% RH.

Shelf life stability measurements for the unencapsulated devices were conducted (Figure 6a). The LD/3D devices based on 4 mg/mL AlmC maintained 93% of their initial efficiency after 91 days of exposure. Under the same aging conditions, only 54% of the initial PCE for the control device remained. The 1 and 2 mg/mL devices also had slower rates of PCE decrease than the control. Figure 6b shows the average rate of normalized PCE decrease obtained from a linear curve fitting to the data in Figure 6a. The gradient is highest for the control and is lowest for the 2 and 4 mg/mL systems. We used the linear fittings of the data in (a) to estimate the times at which the PCE would fall to 80% of the initial value (*t*_80_). As shown in Figure 6c, the *t*_80_ values increase with increasing AImC concentration and are the highest and lowest, respectively, for the 4 mg/mL (212 days) and control (43 days) devices.

The thermal stability of devices was also measured at 85 °C (Figure S19(a)), and it can be seen that the rate of PCE decrease was lowest for the systems prepared at higher AImC concentrations. The *t*_80_ values at 85 °C increase with AImC concentration from 35 h for the control to 132 h for the 4 mg/mL system (Figure S19(b)). Hence, AImC greatly increased the thermal stability of the devices. Photographs of the degradation progressions for perovskite films were obtained in a high-humidity (90% RH) environment as a function of time (Figure 6d). As the AImC concentration increased, the time taken for the films to become bleached increased. The 4 mg/mL solution showed no detectable bleaching after 11 days. Hence, the stability to humidity increased with AImC concentration and is highest for 4 mg/mL system. This trend is congruent with the contact angle data from Figure 2c,d. Accordingly, the progressively hydrophobic surface becomes increasingly effective at opposing moisture ingress, which contributes to the shelf life (Figure 6a) and thermal stability (Figure S19) for the AImC-containing systems. Therefore, AImC provides an increasingly effective barrier as the concentration increases, which slows degradation processes within the LD/3D perovskite films and devices.

## Conclusions

In this study, we introduced a new neutral imidazole additive (AImC) for post-treatment of 3D (Cs_0.05_(FA_0.98_MA_0.02_)_0.95_Pb(I_0.98_Br_0.02_)_3_) perovskite films. AImC had the dual role of a solvent and reactant for the 3D perovskite and triggered the reconstruction of a LD capping layer, which increased the PCE and stability. The finding that AImC remained on the surface is key to its unique reconstruction properties. AImC brings together advantages for smooth surface reconstruction previously reported for MA^30^ and reactive reconstruction associated with LD formation. AImC increased the surface hydrophobicity, which contributed to the pronounced stability improvement of the films and devices. AImC strongly interacts with Pb^2+^ as judged by XRD, FTIR, and XPS data. This neutral additive also strongly passivated the perovskite and decreased the defect density. Due to the increase of FF and *V*_oc_, the 1 mg/mL LD/3D PSC achieved a maximum PCE of 21.42%, which is much higher than the 20.27% for the control device. Because of the stability provided by the LD perovskite layer, the unencapsulated AImC-treated device (4 mg/mL) had a shelf life stability with a projected *t*_80_ of 211 days, which was far superior to 43 days for the control. Our hypothesis that a neutral imidazole liquid could provide reconstruction was upheld by the results for AImC. Accordingly, neutral amine liquids that have the correct solvating and ligating properties (cf. AImC) have good potential to further improve the PCE and stability of PSCs.

## Supporting Information Available

Discussion of identification of LD perovskite dimensionality; AFM images of the films; cross-sectional SEM images and thickness data; data for AImC and IPA evaporation; FTIR spectra of AImC during heating; effects of AImC on the 3D perovskite surface; results of direct reaction of AImC with dispersed perovskite; depiction of LD reconstruction mechanism; tube inversion data showing viscosity differences of IPA and AImC; FTIR spectra; Pb 4f XPS core level spectra; UV–visible spectra; Tauc plots; maximum PL intensity plots; UV–visible and PL spectra of thinner films and wide wavelength region; X-ray patterns and SEM for perovskite prepared using 7 mg/mL AImC; variation of average PCE with FF, EQE data, Kevin probe force microscopy data, Suns-*V*_oc_ and Suns-*J*_sc_ data, space-charge limiting current data, and thermal stability device data; table of photovoltaic parameters; and table of TRPL spectra fitting parameters and references (PDF)

## References

[ref1] Park J.; Kim J.; Yun H.-S.; Paik M. J.; Noh E.; Mun H. J.; Kim M. G.; Shin T. J.; Seok S. I. Controlled growth of perovskite layers with volatile alkylammonium chlorides. Nature 2023, 616, 724–730. 10.1038/s41586-023-05825-y. 36796426

[ref2] Kim H.-S.; Lee C.-R.; Im J.-H.; Lee K.-B.; Moehl T.; Marchioro A.; Moon S.-J.; Humphry-Baker R.; Yum J.-H.; Moser J. E.; et al. Lead iodide perovskite sensitized all-solid-state submicron thin film mesoscopic solar cell with efficiency exceeding 9%. Sci. Rep. 2012, 2, 591 10.1038/srep00591. 22912919 PMC3423636

[ref3] Best Research-Cell Efficiency Chart. https://www.nrel.gov/pv/cell-efficiency.html. (accessed September 12, 2024).

[ref4] Xu W.; Hart L. J. F.; Moss B.; Caprioglio P.; Macdonald T. J.; Furlan F.; Panidi J.; Oliver R. D. J.; Pacalaj R. A.; Heeney M.; Gasparini N.; Snaith H. J.; Barnes P. R. F.; Durrant J. R. Impact of Interface Energetic Alignment and Mobile Ions on Charge Carrier Accumulation and Extraction in p-i-n Perovskite Solar Cells. Adv. Energy Mater. 2023, 13, 2301102 10.1002/aenm.202301102.

[ref5] Kim G.; Min H.; Lee K. S.; Lee D. Y.; Yoon S. M.; Seok S. I. Impact of strain relaxation on performance of α-formamidinium lead iodide perovskite solar cells. Science 2020, 370, 108–112. 10.1126/science.abc4417. 33004518

[ref6] Yoo J. J.; Seo G.; Chua M. R.; Park T. G.; Lu Y.; Rotermund F.; Kim Y.-K.; Moon C. S.; Jeon N. J.; Correa-Baena J.-P.; Bulović V.; Shin S. S.; Bawendi M. G.; Seo J. Efficient perovskite solar cells via improved carrier management. Nature 2021, 590, 587–593. 10.1038/s41586-021-03285-w. 33627807

[ref7] Zhang W.; Pathak S.; Sakai N.; Stergiopoulos T.; Nayak P. K.; Noel N. K.; Haghighirad A. A.; Burlakov V. M.; Dequilettes D. W.; Sadhanala A.; et al. Enhanced optoelectronic quality of perovskite thin films with hypophosphorous acid for planar heterojunction solar cells. Nat. Commun. 2015, 6, 10030 10.1038/ncomms10030. 26615763 PMC4674686

[ref8] Su H.; Zhang J.; Hu Y.; Du X.; Yang Y.; You J.; Gao L.; Liu S. Fluoroethylamine Engineering for effective passivation to attain 23.4% efficiency perovskite solar cells with superior stability. Adv. Energy Mater. 2021, 11, 2101454 10.1002/aenm.202101454.

[ref9] Azmi R.; Ugur E.; Seitkhan A.; Aljamaan F.; Subbiah A. S.; Liu J.; Harrison G. T.; Nugraha M. I.; Eswaran M. K.; Babics M.; Chen Y.; Xu F.; Allen T. G.; Rehman Au.; Wang C.-L.; Anthopoulos T. D.; Schwingenschlögl U.; De Bastiani M.; Aydin E.; De Wolf S. Damp heat–stable perovskite solar cells with tailored-dimensionality 2D/3D heterojunctions. Science 2022, 376, 73–77. 10.1126/science.abm5784. 35175829

[ref10] Luo L.; Zeng H.; Wang Z.; Li M.; You S.; Chen B.; Maxwell A.; An Q.; Cui L.; Luo D.; Hu J.; Li S.; Cai X.; Li W.; Li L.; Guo R.; Huang R.; Liang W.; Lu Z.-H.; Mai L.; Rong Y.; Sargent E. H.; Li X. Stabilization of 3D/2D perovskite heterostructures via inhibition of ion diffusion by cross-linked polymers for solar cells with improved performance. Nat. Energy 2023, 8, 294–303. 10.1038/s41560-023-01205-y.

[ref11] Li C.; Zhu R.; Yang Z.; Lai J.; Tan J.; Luo Y.; Ye S. Boosting Charge Transport in a 2D/3D Perovskite Heterostructure by Selecting an Ordered 2D Perovskite as the Passivator. Angew. Chem., Int. Ed. 2023, 62, e202214208 10.1002/anie.202214208. 36470848

[ref12] Hou S.; Ma Z.; Li Y.; Du Z.; Chen Y.; Yang J.; You W.; Yang Q.; Yu T.; Huang Z.; Li G.; Wang H.; Liu Q.; Yan G.; Li H.; Huang Y.; Zhang W.; Abdi-Jalebi M.; Ou Z.; Sun K.; Su R.; Long W. Bulk In Situ Reconstruction of Heterojunction Perovskite Enabling Stable Solar Cells Over 24% Efficiency. Adv. Funct. Mater. 2024, 34, 2310133 10.1002/adfm.202310133.

[ref13] Liu Q.; Ou Z.; Ma Z.; Huang Z.; Li Y.; Hou S.; Ren J.; Peng J.; Bai L.; Yu H.; Lv Z.; Xiang Y.; Yu J.; Zhang W.; Jiang F.; Sun K.; Zhu T.; Ding L. Perovskite solar cells with self-disintegrating seeds deliver an 83.64% fill factor. Nano Energy 2024, 127, 109751 10.1016/j.nanoen.2024.109751.

[ref14] Ma C.; Shen D.; Ng T. W.; Lo M. F.; Lee C. S. 2D perovskites with short interlayer distance for high-performance solar cell application. Adv. Mater. 2018, 30, 1800710 10.1002/adma.201800710. 29665101

[ref15] Shen L.; Wu H.; Cao Z.; Zhang X.; Liu L.; Sawwan H.; Zhu T.; Zheng J.; Wang H.; Gong X. Two-Dimensional Metal Halide Perovskites Created by Binary Conjugated Organic Cations for High-Performance Perovskite Photovoltaics. ACS Appl. Mater. Interfaces 2024, 16, 19318–19329. 10.1021/acsami.4c00288. 38577894

[ref16] Kim J.; Hwang T.; Lee B.; Lee S.; Park K.; Park H. H.; Park B. An Aromatic Diamine Molecule as the A-Site Solute for Highly Durable and Efficient Perovskite Solar Cells. Small Methods 2019, 3, 1800361 10.1002/smtd.201800361.

[ref17] Deng C.; Wu J.; Du Y.; Chen Q.; Song Z.; Li G.; Wang X.; Lin J.; Sun W.; Huang M.; et al. Surface reconstruction and in situ formation of 2D layer for efficient and stable 2D/3D perovskite solar cells. Small Methods 2021, 5, 2101000 10.1002/smtd.202101000. 34928027

[ref18] Wang Z.; Lin Q.; Chmiel F. P.; Sakai N.; Herz L. M.; Snaith H. J. Efficient ambient-air-stable solar cells with 2D–3D heterostructured butylammonium-caesium-formamidinium lead halide perovskites. Nat. Energy 2017, 2, 1–10. 10.1038/nenergy.2017.135.

[ref19] Mao L.; Stoumpos C. C.; Kanatzidis M. G. Two-Dimensional Hybrid Halide Perovskites: Principles and Promises. J. Am. Chem. Soc. 2019, 141, 1171–1190. 10.1021/jacs.8b10851. 30399319

[ref20] Zhao X.; Liu T.; Loo Y.-L. Advancing 2D Perovskites for Efficient and Stable Solar Cells: Challenges and Opportunities. Adv. Mater. 2022, 34, 2105849 10.1002/adma.202105849. 34668250

[ref21] Hu F.; Lou Y. H.; Wang Z. K. Functional 2D Phases in Mixed Dimensional Perovskite Photovoltaics. Adv. Funct. Mater. 2023, 33, 2304848 10.1002/adfm.202304848.

[ref22] Lai H.; Lu D.; Xu Z.; Zheng N.; Xie Z.; Liu Y. Organic-Salt-Assisted Crystal Growth and Orientation of Quasi-2D Ruddlesden–Popper Perovskites for Solar Cells with Efficiency over 19%. Adv. Mater. 2020, 32, 2001470 10.1002/adma.202001470. 32627858

[ref23] Byeon J.; Cho S. H.; Jiang J.; Jang J.; Katan C.; Even J.; Xi J.; Choi M.; Lee Y. S. Structural Isomer of Fluorinated Ruddlesden-Popper Perovskites Toward Efficient and Stable 2D/3D Perovskite Solar Cells. ACS Appl. Mater. Interfaces 2023, 15, 27853–27864. 10.1021/acsami.3c01754. 37272377

[ref24] Quan L. N.; Yuan M.; Comin R.; Voznyy O.; Beauregard E. M.; Hoogland S.; Buin A.; Kirmani A. R.; Zhao K.; Amassian A.; Kim D. H.; Sargent E. H. Ligand-Stabilized Reduced-Dimensionality Perovskites. J. Am. Chem. Soc. 2016, 138, 2649–2655. 10.1021/jacs.5b11740. 26841130

[ref25] Lin Y.; Bai Y.; Fang Y.; Chen Z.; Yang S.; Zheng X.; Tang S.; Liu Y.; Zhao J.; Huang J. Enhanced thermal stability in perovskite solar cells by assembling 2D/3D stacking structures. J. Phys. Chem. Lett. 2018, 9, 654–658. 10.1021/acs.jpclett.7b02679. 29350044

[ref26] Bhatnagar A.; Sharma P.; Kumar N. A review on “Imidazoles”: Their chemistry and pharmacological potentials. Int. J. PharmTech Res. 2011, 3, 268–282.

[ref27] Zhang Y.; Park N.-G. Quasi-two-dimensional perovskite solar cells with efficiency exceeding 22%. ACS Energy Lett. 2022, 7, 757–765. 10.1021/acsenergylett.1c02645.

[ref28] Liu G.; Zheng H.; Ye J.; Xu S.; Zhang L.; Xu H.; Liang Z.; Chen X.; Pan X. Mixed-phase low-dimensional perovskite-assisted interfacial lead directional management for stable perovskite solar cells with efficiency over 24%. ACS Energy Lett. 2021, 6, 4395–4404. 10.1021/acsenergylett.1c01878.

[ref29] Wang Y.; Song J.; Ye J.; Jin Y.; Yin X.; Su Z.; Hu L.; Wu Y.; Qiu C.; Wang H.; Yan W.; Li Z. Surface termination passivation of imidazole-based diiodide enabling efficient inverted perovskite solar cells. Chem. Commun. 2023, 59, 6580–6583. 10.1039/D3CC01379K. 37183488

[ref30] Zhou Z.; Wang Z.; Zhou Y.; Pang S.; Wang D.; Xu H.; Liu Z.; Padture N. P.; Cui G. Methylamine-gas-induced defect-healing behavior of CH3NH3PbI3 thin films for perovskite solar cells. Angew. Chem., Int. Ed. 2015, 127, 9841–9845. 10.1002/ange.201504379. 26118666

[ref31] Chen P.; Bai Y.; Wang S.; Lyu M.; Yun J. H.; Wang L. In situ growth of 2D perovskite capping layer for stable and efficient perovskite solar cells. Adv. Funct. Mater. 2018, 28, 1706923 10.1002/adfm.201706923.

[ref32] Sun J.; Zhang X.; Ling X.; Yang Y.; Wang Y.; Guo J.; Liu S. F.; Yuan J.; Ma W. A penetrated 2D/3D hybrid heterojunction for high-performance perovskite solar cells. J. Mater. Chem. A 2021, 9, 23019–23027. 10.1039/D1TA06514A.

[ref33] Niu T.; Xie Y. M.; Xue Q.; Xun S.; Yao Q.; Zhen F.; Yan W.; Li H.; Brédas J. L.; Yip H. L.; Cao Y. Spacer engineering of diammonium-based 2D perovskites toward efficient and stable 2D/3D heterostructure perovskite solar cells. Adv. Energy Mater. 2022, 12, 2102973 10.1002/aenm.202102973.

[ref34] Park B.-w.; Kwon H. W.; Lee Y.; Lee D. Y.; Kim M. G.; Kim G.; Kim K.-j.; Kim Y. K.; Im J.; Shin T. J.; Seok S. I. Stabilization of formamidinium lead triiodide α-phase with isopropylammonium chloride for perovskite solar cells. Nat. Energy 2021, 6, 419–428. 10.1038/s41560-021-00802-z.

[ref35] Zhou Q.; Liang L.; Hu J.; Cao B.; Yang L.; Wu T.; Li X.; Zhang B.; Gao P. High-performance perovskite solar cells with enhanced environmental stability based on a (p-FC6H4C2H4NH3) 2 [PbI4] capping layer. Adv. Energy Mater. 2019, 9, 1802595 10.1002/aenm.201802595.

[ref36] Kim Y. J.; Park C. R. Analysis of Problematic Complexing Behavior of Ferric Chloride with N,N-Dimethylformamide Using Combined Techniques of FT-IR, XPS, and TGA/DTG. Inorg. Chem. 2002, 41, 6211–6216. 10.1021/ic011306p. 12444762

[ref37] Jiang Q.; Zhao Y.; Zhang X.; Yang X.; Chen Y.; Chu Z.; Ye Q.; Li X.; Yin Z.; You J. Surface passivation of perovskite film for efficient solar cells. Nat. Photonics 2019, 13, 460–466. 10.1038/s41566-019-0398-2.

[ref38] Kowoll T.; Müller E.; Fritsch-Decker S.; Hettler S.; Störmer H.; Weiss C.; Gerthsen D. Contrast of backscattered electron SEM images of nanoparticles on substrates with complex structure. Scanning 2017, 2017, 4907457 10.1155/2017/4907457. 29109816 PMC5661778

[ref39] Goldstein J. I. Scanning Electron Microscopy and X-ray Microanalysis: A Text Book for Biologists, Materials Scientists and Geoligists.; Plenum Press: New York, 1992.

[ref40] Elschner T.; Bračič M.; Mohan T.; Kargl R.; Stana Kleinschek K. Modification of cellulose thin films with lysine moieties: a promising approach to achieve antifouling performance. Cellulose 2018, 25, 537–547. 10.1007/s10570-017-1538-9.

[ref41] Yoo J. J.; Wieghold S.; Sponseller M. C.; Chua M. R.; Bertram S. N.; Hartono N. T. P.; Tresback J. S.; Hansen E. C.; Correa-Baena J.-P.; Bulović V.; Buonassisi T.; Shin S. S.; Bawendi M. G. An interface stabilized perovskite solar cell with high stabilized efficiency and low voltage loss. Energy Environ. Sci. 2019, 12, 2192–2199. 10.1039/C9EE00751B.

[ref42] Liu Z.; Meng K.; Wang X.; Qiao Z.; Xu Q.; Li S.; Cheng L.; Li Z.; Chen G. In situ observation of vapor-assisted 2D–3D heterostructure formation for stable and efficient perovskite solar cells. Nano Lett. 2020, 20, 1296–1304. 10.1021/acs.nanolett.9b04759. 31986053

[ref43] Hou X.; Huang S.; Ou-Yang W.; Pan L.; Sun Z.; Chen X. Constructing efficient and stable perovskite solar cells via interconnecting perovskite grains. ACS Appl. Mater. Interfaces 2017, 9, 35200–35208. 10.1021/acsami.7b08488. 28936870

[ref44] Ruiz D. S.; Cristobal P. A.; Laurenti M.; Retama J. R.; Lopez-Cabarcos E. Polymer Diffusion in Microgels with Upper Critical Solution Temperature as Studied by Incoherent Neutron Scattering. J. Phys.: Conf. Ser. 2014, 549, 012012 10.1088/1742-6596/549/1/012012.

[ref45] Shimanouchi T.; Matsuura H.; Ogawa Y.; Harada I. Tables of molecular vibrational frequencies. J. Phys. Chem. Ref. Data 1978, 7, 1323–1444. 10.1063/1.555587.

[ref46] Jacox M. E. Vibrational and electronic energy levels of polyatomic transient molecules. Supplement B. J. Phys. Chem. Ref. Data 2003, 32, 1–441. 10.1063/1.1497629.

[ref47] Wang H.; Wang J.; He Q.; Chang J.; Chen S.; Zhong C.; Wu M.; Zhao X.; Chen H.; Tian Q.; Li M.; Lai J.; Yang Y.; Li R.; Wu B.; Huang W.; Qin T.; Wang F. Interface Dipole Management of D–A-Type Molecules for Efficient Perovskite Solar Cells. Angew. Chem., Int. Ed. 2024, 63, e202404289 10.1002/anie.202404289. 38712497

[ref48] Tan S.; Huang T.; Yavuz I.; Wang R.; Weber M. H.; Zhao Y.; Abdelsamie M.; Liao M. E.; Wang H.-C.; Huynh K.; et al. Surface reconstruction of halide perovskites during post-treatment. J. Am. Chem. Soc. 2021, 143, 6781–6786. 10.1021/jacs.1c00757. 33915050

[ref49] Zheng J.; Chen J.; Ouyang D.; Huang Z.; He X.; Kim J.; Choy W. C. Critical role of functional groups in defect passivation and energy band modulation in efficient and stable inverted perovskite solar cells exceeding 21% efficiency. ACS Appl. Mater. Interfaces 2020, 12, 57165–57173. 10.1021/acsami.0c18862. 33296167

[ref50] Li G.; Song J.; Wu J.; Song Z.; Wang X.; Sun W.; Fan L.; Lin J.; Huang M.; Lan Z.; Gao P. Efficient and stable 2D@ 3D/2D perovskite solar cells based on dual optimization of grain boundary and interface. ACS Energy Lett. 2021, 6, 3614–3623. 10.1021/acsenergylett.1c01649.

[ref51] Lee D.-K.; Lim K.-S.; Lee J.-W.; Park N.-G. Scalable perovskite coating via anti-solvent-free Lewis acid–base adduct engineering for efficient perovskite solar modules. J. Mater. Chem. A 2021, 9, 3018–3028. 10.1039/D0TA10366G.

[ref52] Lee J.-W.; Dai Z.; Lee C.; Lee H. M.; Han T.-H.; De Marco N.; Lin O.; Choi C. S.; Dunn B.; Koh J.; et al. Tuning molecular interactions for highly reproducible and efficient formamidinium perovskite solar cells via adduct approach. J. Am. Chem. Soc. 2018, 140, 6317–6324. 10.1021/jacs.8b01037. 29723475

[ref53] Lee J.-W.; Dai Z.; Han T.-H.; Choi C.; Chang S.-Y.; Lee S.-J.; De Marco N.; Zhao H.; Sun P.; Huang Y.; Yang Y. 2D perovskite stabilized phase-pure formamidinium perovskite solar cells. Nat. Commun. 2018, 9, 3021 10.1038/s41467-018-05454-4. 30069012 PMC6070510

[ref54] Li H.; Shi J.; Deng J.; Chen Z.; Li Y.; Zhao W.; Wu J.; Wu H.; Luo Y.; Li D. Intermolecular π–π conjugation self-assembly to stabilize surface passivation of highly efficient perovskite solar cells. Adv. Mater. 2020, 32, 1907396 10.1002/adma.201907396. 32350937

[ref55] Guo J.; Wang B.; Lu D.; Wang T.; Liu T.; Wang R.; Dong X.; Zhou T.; Zheng N.; Fu Q.; Xie Z.; Wan X.; Xing G.; Chen Y.; Liu Y. Ultralong Carrier Lifetime Exceeding 20 μs in Lead Halide Perovskite Film Enable Efficient Solar Cells. Adv. Mater. 2023, 35, 2212126 10.1002/adma.202212126. 37163976

[ref56] Li X.; Li W.; Yang Y.; Lai X.; Su Q.; Wu D.; Li G.; Wang K.; Chen S.; Sun X. W.; Kyaw A. K. K. Defects Passivation With Dithienobenzodithiophene-based π-conjugated Polymer for Enhanced Performance of Perovskite Solar Cells. Sol. RRL 2019, 3, 1900029 10.1002/solr.201900029.

[ref57] Zhu Z.; Bai Y.; Lee H. K. H.; Mu C.; Zhang T.; Zhang L.; Wang J.; Yan H.; So S. K.; Yang S. Polyfluorene derivatives are high-performance organic hole-transporting materials for inorganic– organic hybrid perovskite solar cells. Adv. Funct. Mater. 2014, 24, 7357–7365. 10.1002/adfm.201401557.

[ref58] Jiang J.; Tian C.; Zhang Z.; Liu X. X.; Wang X.; Zheng Y.; Zhang Z.; Wang L.; Wu X.; Liang J.; Chen C. C. Mixed dimensionality of 2D/3D heterojunctions for improving charge transport and long-term stability in high-efficiency 1.63 eV bandgap perovskite solar cells. Mater. Adv. 2022, 3, 5786–5795. 10.1039/D2MA00391K.

[ref59] De Marco N.; Zhou H.; Chen Q.; Sun P.; Liu Z.; Meng L.; Yao E.-P.; Liu Y.; Schiffer A.; Yang Y. Guanidinium: a route to enhanced carrier lifetime and open-circuit voltage in hybrid perovskite solar cells. Nano Lett. 2016, 16, 1009–1016. 10.1021/acs.nanolett.5b04060. 26790037

[ref60] Proppe A. H.; Wei M.; Chen B.; Quintero-Bermudez R.; Kelley S. O.; Sargent E. H. Photochemically cross-linked quantum well ligands for 2D/3D perovskite photovoltaics with improved photovoltage and stability. J. Am. Chem. Soc. 2019, 141, 14180–14189. 10.1021/jacs.9b05083. 31422664

[ref61] Li P.; Zhang Y.; Liang C.; Xing G.; Liu X.; Li F.; Liu X.; Hu X.; Shao G.; Song Y. Phase pure 2D perovskite for high-performance 2D–3D heterostructured perovskite solar cells. Adv. Mater. 2018, 30, 1805323 10.1002/adma.201805323. 30387210

[ref62] Sutanto A. A.; Caprioglio P.; Drigo N.; Hofstetter Y. J.; Garcia-Benito I.; Queloz V. I.; Neher D.; Nazeeruddin M. K.; Stolterfoht M.; Vaynzof Y.; Grancini G. 2D/3D perovskite engineering eliminates interfacial recombination losses in hybrid perovskite solar cells. Chem 2021, 7, 1903–1916. 10.1016/j.chempr.2021.04.002.

[ref63] von Hauff E. 2D or not 2D: Eliminating interfacial losses in perovskite solar cells. Chem 2021, 7, 1694–1696. 10.1016/j.chempr.2021.06.020.

[ref64] Zhang B.; Gao D.; Li M.; Shang X.; Li Y.; Chen C.; Pauporté T. Heterojunction In Situ Constructed by a Novel Amino Acid-Based Organic Spacer for Efficient and Stable Perovskite Solar Cells. ACS Appl. Mater. Interfaces 2022, 14, 40902–40912. 10.1021/acsami.2c09926. 36054908

[ref65] Glowienka D.; Galagan Y. Light intensity analysis of photovoltaic parameters for perovskite solar cells. Adv. Mater. 2022, 34, 2105920 10.1002/adma.202105920. 34676926 PMC11469270

[ref66] Cowan S. R.; Roy A.; Heeger A. J. Recombination in polymer-fullerene bulk heterojunction solar cells. Phys. Rev. B 2010, 82, 245207 10.1103/PhysRevB.82.245207.

[ref67] Tress W.; Yavari M.; Domanski K.; Yadav P.; Niesen B.; Baena J. P. C.; Hagfeldt A.; Graetzel M. Interpretation and evolution of open-circuit voltage, recombination, ideality factor and subgap defect states during reversible light-soaking and irreversible degradation of perovskite solar cells. Energy Environ. Sci. 2018, 11, 151–165. 10.1039/C7EE02415K.

[ref68] Kirchartz T.; Nelson J. Meaning of reaction orders in polymer: fullerene solar cells. Phys. Rev. B 2012, 86, 165201 10.1103/PhysRevB.86.165201.

[ref69] Li Y.; Liu L.; Zheng C.; Liu Z.; Chen L.; Yuan N.; Ding J.; Wang D.; Liu S. Plant-Derived l-Theanine for Ultraviolet/Ozone Resistant Perovskite Photovoltaics. Adv. Energy Mater. 2023, 13, 2203190 10.1002/aenm.202203190.

[ref70] Choi H.; Liu X.; Kim H. I.; Kim D.; Park T.; Song S. A facile surface passivation enables thermally stable and efficient planar perovskite solar cells using a novel IDTT-based small molecule additive. Adv. Energy Mater. 2021, 11, 2003829 10.1002/aenm.202003829.

[ref71] Huang Y.; Li L.; Liu Z.; Jiao H.; He Y.; Wang X.; Zhu R.; Wang D.; Sun J.; Chen Q.; Zhou H. The intrinsic properties of FA(1–x)MAxPbI3 perovskite single crystals. J. Mater. Chem. A 2017, 5, 8537–8544. 10.1039/C7TA01441D.

[ref72] Han Q.; Bae S.-H.; Sun P.; Hsieh Y.-T.; Yang Y.; Rim Y. S.; Zhao H.; Chen Q.; Shi W.; Li G.; Yang Y. Single Crystal Formamidinium Lead Iodide (FAPbI3): Insight into the Structural, Optical, and Electrical Properties. Adv. Mater. 2016, 28, 2253–2258. 10.1002/adma.201505002. 26790006

