# Supplementary material for: Neutral Ligand Triggered Low-Dimensional Reconstruction for Improving the Efficiency and Stability of Perovskite Solar Cells
Source: ACS Appl Energy Mater. 2024 Oct 18;7(21):9723–34. doi: 10.1021/acsaem.4c01301 (PMC11558562; doi:10.1021/acsaem.4c01301)
Supplement: Supplementary file 1 — ae4c01301_si_001.pdf [file ae4c01301_si_001.pdf]

SUPPORTING INFORMATION for

Neutral ligand triggered low-dimensional reconstruction for improving the efficiency and stability of perovskite solar cells

Ran Wang[a, b †, *], Zhenyu Jia[a, †, *], Ben F. Spencer[a,c], Dawei Zhao[c], Andrew G. Thomas[c], Osama M. Alkhudhari[a,d], David J. Lewis[a], Robert J. Cernik[a], Ashwaq Alanazi[a] and Brian R. Saunders[a,*]

a) *Department of Materials, University of Manchester, Engineering Building A, Manchester, M1 7HL, U. K.*

b) *Center for Micro-Nano Systems, School of Information Science and Technology (SIST), Fudan University, Shanghai, 200433 P. R. China*

c) *Photon Science Institute, the Henry Royce Institute, University of Manchester, Manchester, M13 9PL, U.K.*

d) *Department of Chemistry, College of Science, Taif University, Taif 21944, Saudi Arabia.*

Corresponding authors:

Ran Wang: ran.wang-4@manchester.ac.uk

Zhenyu Jia: zhenyu.jia@postgrad.manchester.ac.uk

Brian R. Saunders: brian.saunders@machester.ac.uk

[†] These authors contributed equally to the work

Additional note 1: Evidence for 2D perovskite dimensionality for 1 to 4 mg/mL films

To identify the value of n for our LD $(A')_2(A)_{n-1}Pb_nX_{3n+1}$ perovskites prepared using 1 mg/mL to 4 mg/mL AImC, we assumed that the unique scattering peak at 2θ = 7.9° in Figure 3(a) is due to a first-order reflection (m = 1) and obeyed Bragg's law. (Note that we use m instead of n in the Bragg equation to avoid confusion with the parameter n used in the perovskite structure.)

$$m\lambda = 2d sin\theta \qquad \text{(S1)}$$

where λ is the X-ray wavelength (1.54 Å).

Accordingly, the distance between planes (d) for such an m = 1 reflection is given by

$$d = \frac{\lambda}{2sin\theta} \qquad \text{(S2)}$$

and, hence, d = 11.2 Å.

We next estimate the value of 2θ for a second-order reflection (m = 2) using the following equation.

$$2\theta = 2sin^{-1}\left(\frac{m\lambda}{2d}\right) \qquad \text{(S3)}$$

Using the values of d, m and λ above, Equation S3 returns a 2θ value of 15.9°. Scrutiny of the expanded XRD pattern in Figure 3(b) shows a *unique peak at exactly 2θ = 15.9°* which is not present in the control. This unique peak is therefore assigned as the second-order reflection from the LD phase identified above.

We next assume that the layer thickness (d) corresponds to the $d_{(001)}$ spacing and that the following equation applies[1]

$$d_{(001)} = a^{*}n + x \qquad \text{(S4)}$$

where a^* is the lattice parameter and is the length of PbI_6^{4-}. The latter is taken as[2] 6.3 Å. The value of x is the thickness of the complete AImC layer. The value for n in Equation S4 is the number of octahedral inorganic sheet layers. Because $d_{(001)}$ = 11.2 Å, it follows from Equation S4 that there is a distance of about 5 Å for the spacer ligands to occupy. Consequently, any value of n greater than 1 is not physically reasonable because there would not be enough space to accommodate more layers. Accordingly, the value for n is tentatively assigned as unity. We note that whilst these data and analysis provide some evidence for the presence of 2D perovskite, they do not unequivocally prove that 2D perovskite was present.

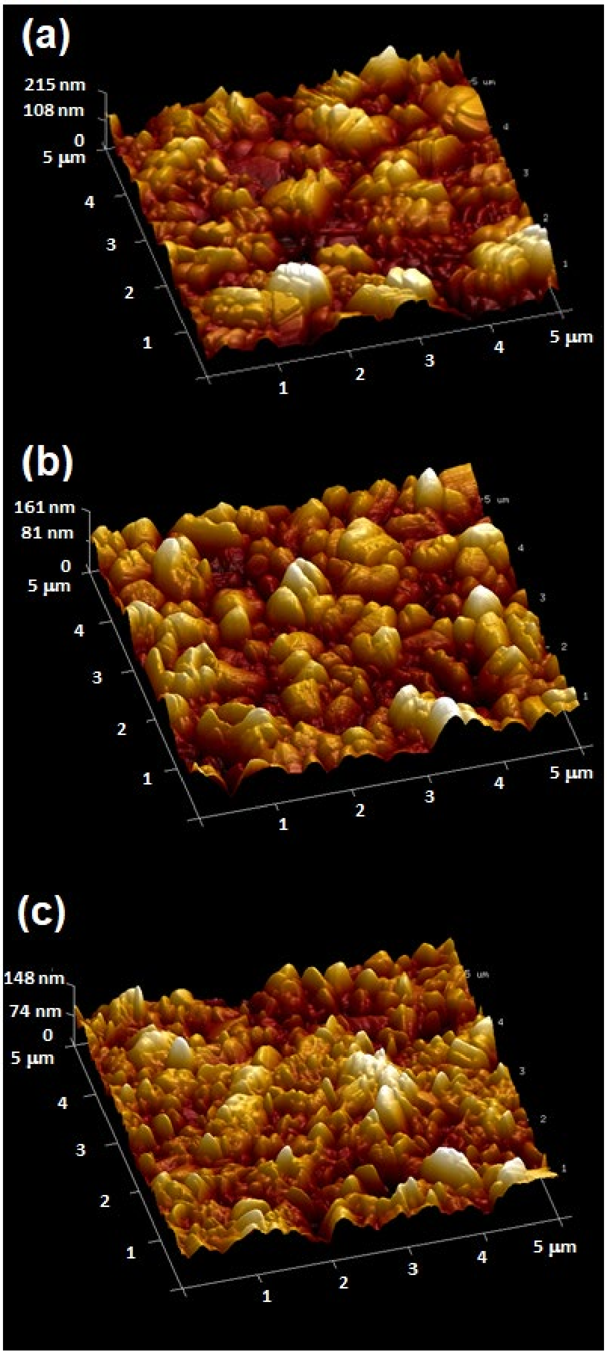

Figure S1. AFM images of **(a)** control, **(b)** 1 mg/mL and **(c)** 4 mg/mL post-treated perovskite films.

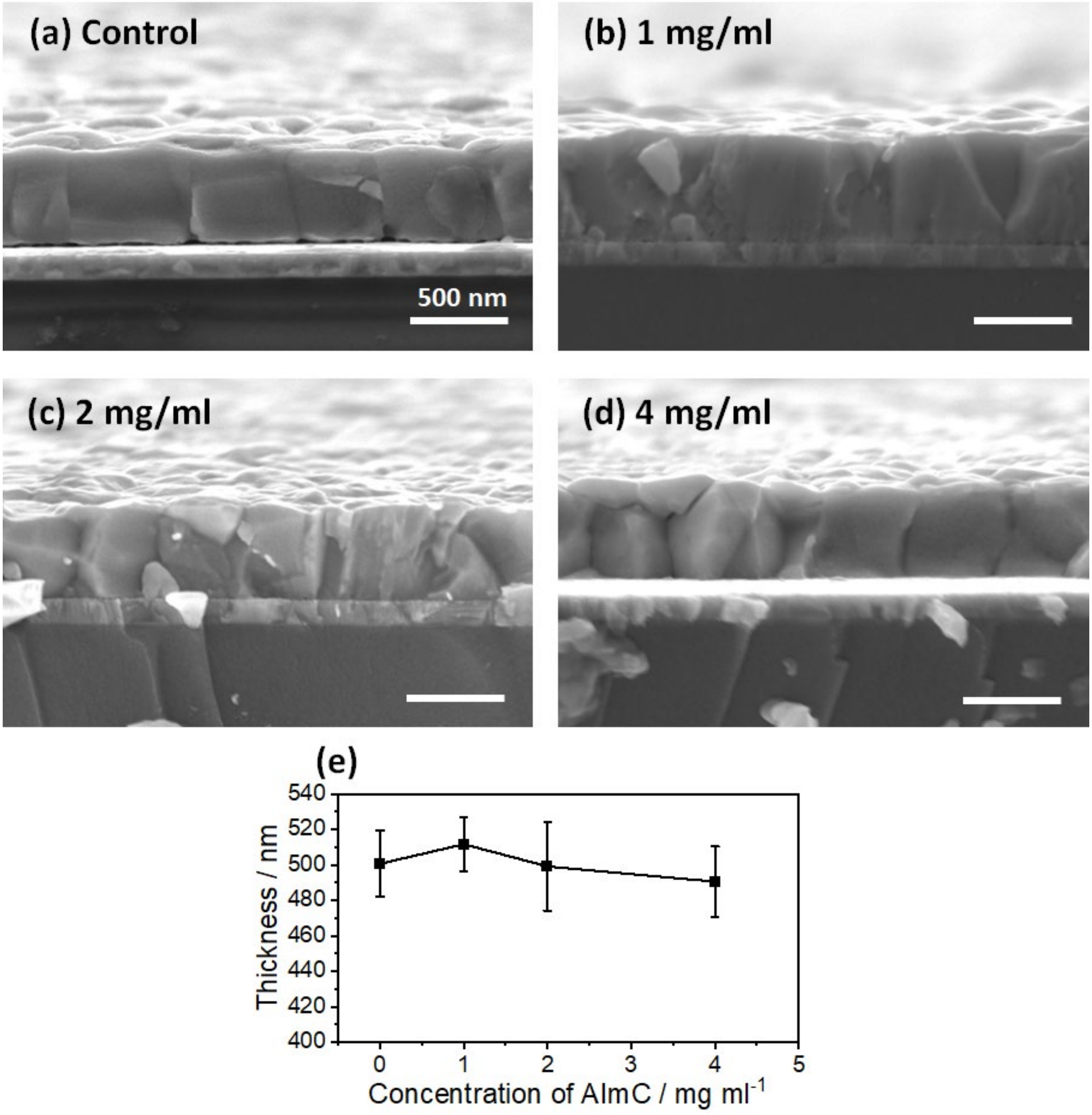

Figure S2. **(a) - (d)** Cross-sectional SEM images of the perovskite films. The AImC concentration used is shown. **(e)** Thickness of the LD/3D films as a function of AImC concentration.

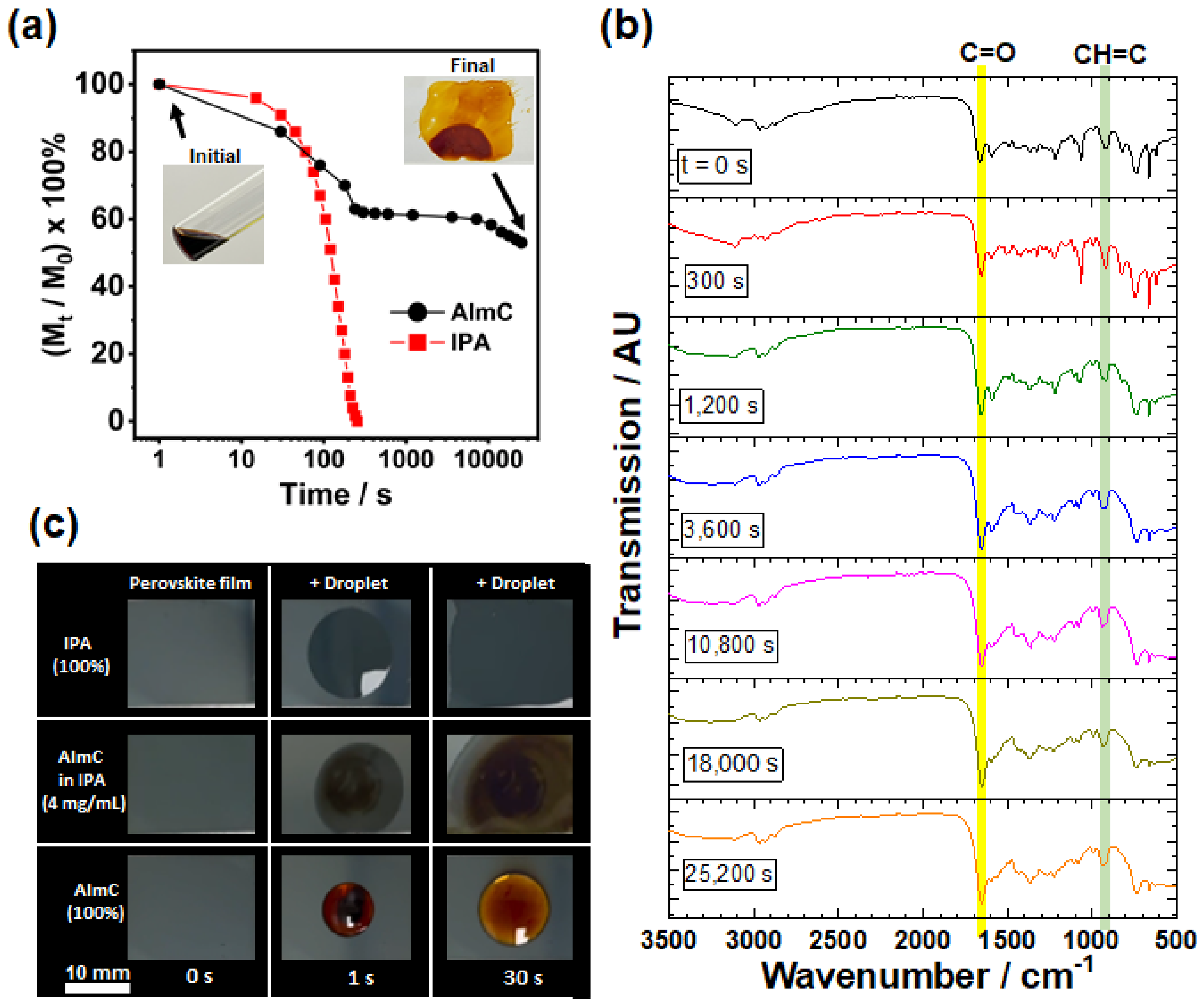

Figure S3. (a) Relative mass of IPA and AImC as a function of time during evaporation at 100 °C. The inset shows a photograph of the final AImC sample. M_t is the mass of liquid at time t, while M_0 is initial mass of liquid. **(b)** FTIR spectra measured for AImC heated at 100 °C for various times (shown). **(c)** Photographs showing the effects of IPA, AImC + IPA and AImC on the surface of the 3D perovskite film as a function of time. This experiment was conducted at room temperature.

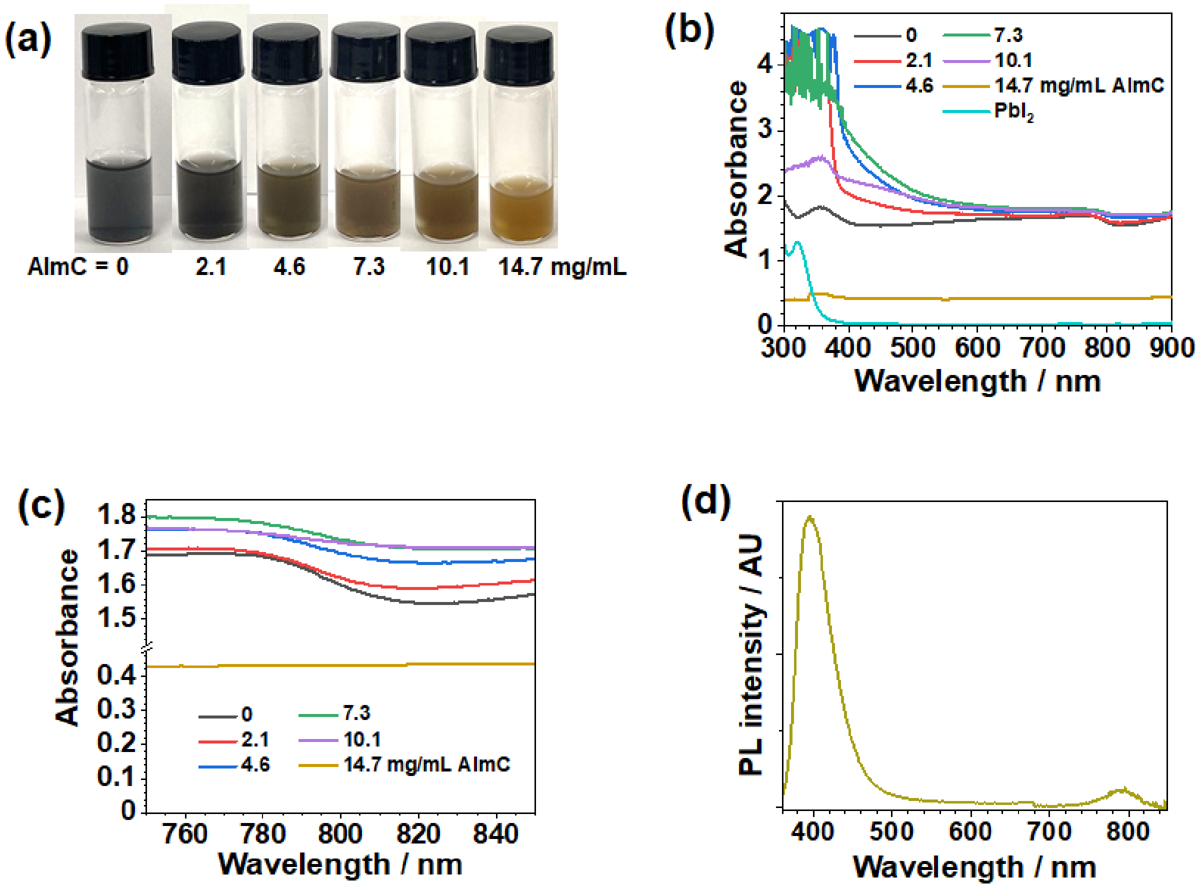

Figure S4. (a) Photographs of 3D perovskite that had been scrapped off the substrate and redispersed in IPA and various concentrations of AImC added. This experiment was performed at room temperature. **(b)** UV-visible spectra recorded for the dispersions from (a). A spectrum for PbI_2 in DMF/DMSO is also shown. **(c)** Expanded view of the high wavelength region from (b). **(d)** PL spectrum of the dispersion treated with 14.7 mg/mL AImC.

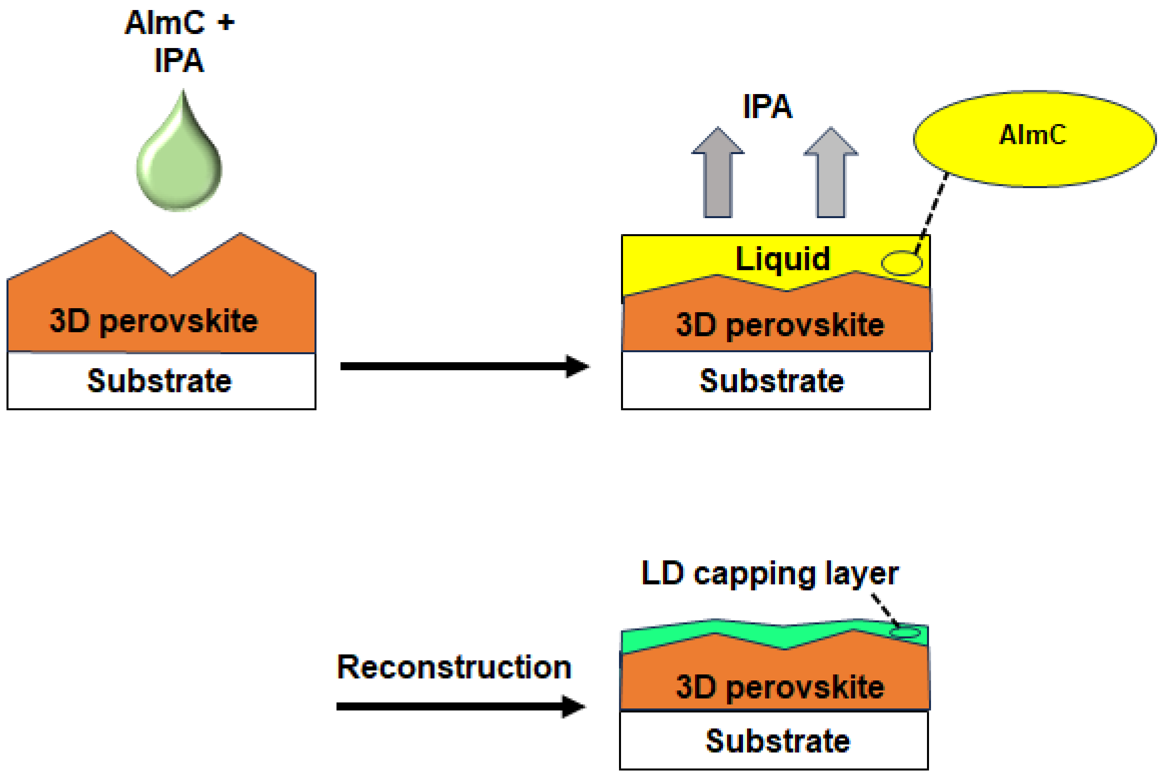

Figure S5. Proposed AImC-triggered LD reconstruction mechanism for the perovskite films in this study. The reconstructed LD capping layer is less rough than the original 3D surface.

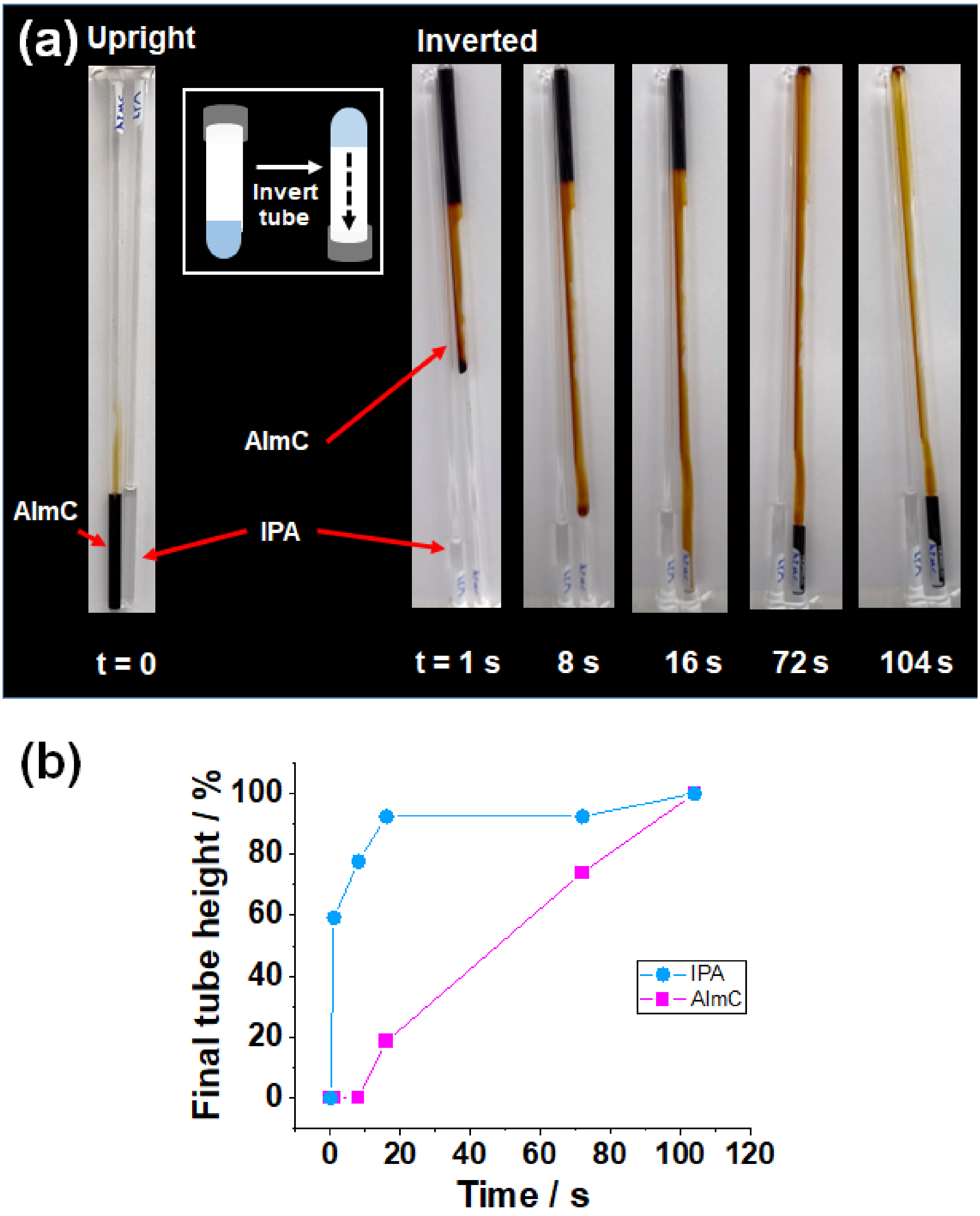

Figure S6. **(a)** Photographs of two NMR tubes containing equal volumes of AImC and IPA before and after inversion. The AImC takes longer to flow to the bottom of the tube because it is more viscous. **(b)** Relative heights of the liquids at the bottom of the tubes as a function of time.

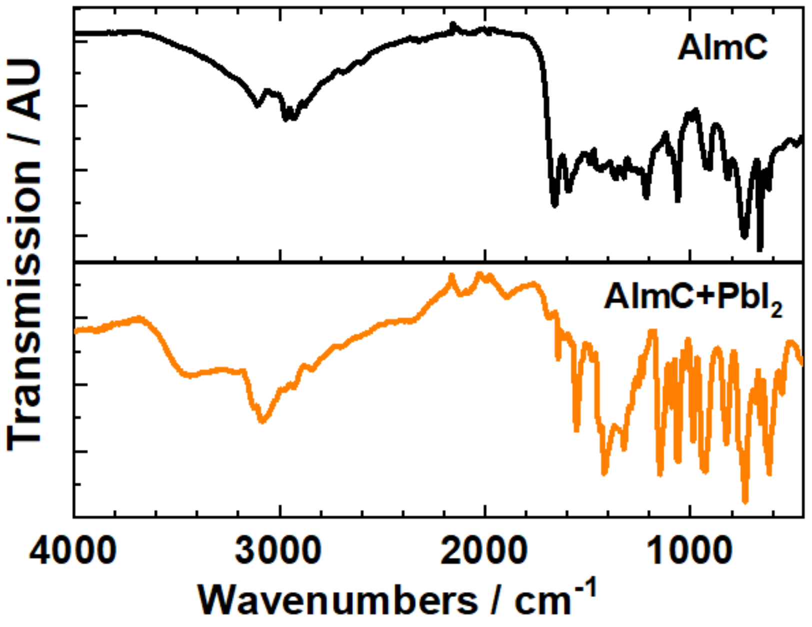

Figure S7. FTIR spectra of AImC and a mixture of AImC/PbI_2.

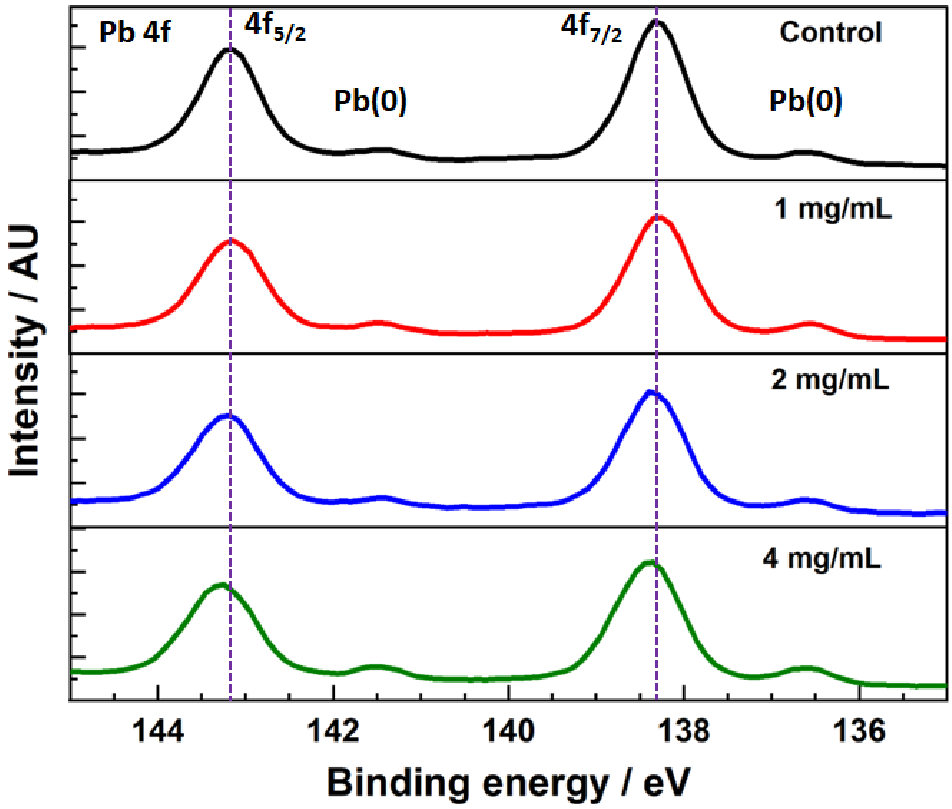

Figure S8. XPS spectra for Pb 4f core levels of the perovskite films. The AImC concentrations used are shown.

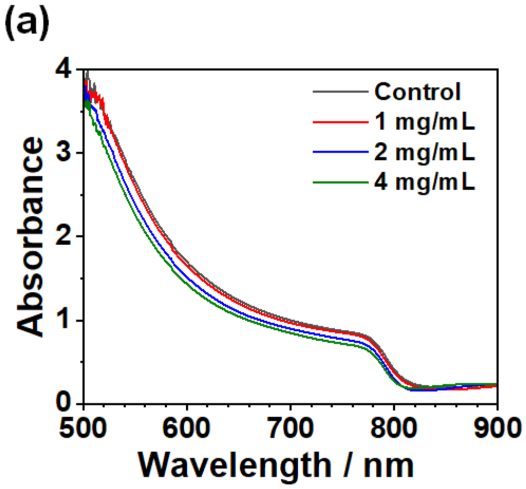

Figure S9. (a) UV-visible absorption spectra of control film and AImC-treated LD/3D films. **(b)** Expanded view of the high wavelength region from (a).

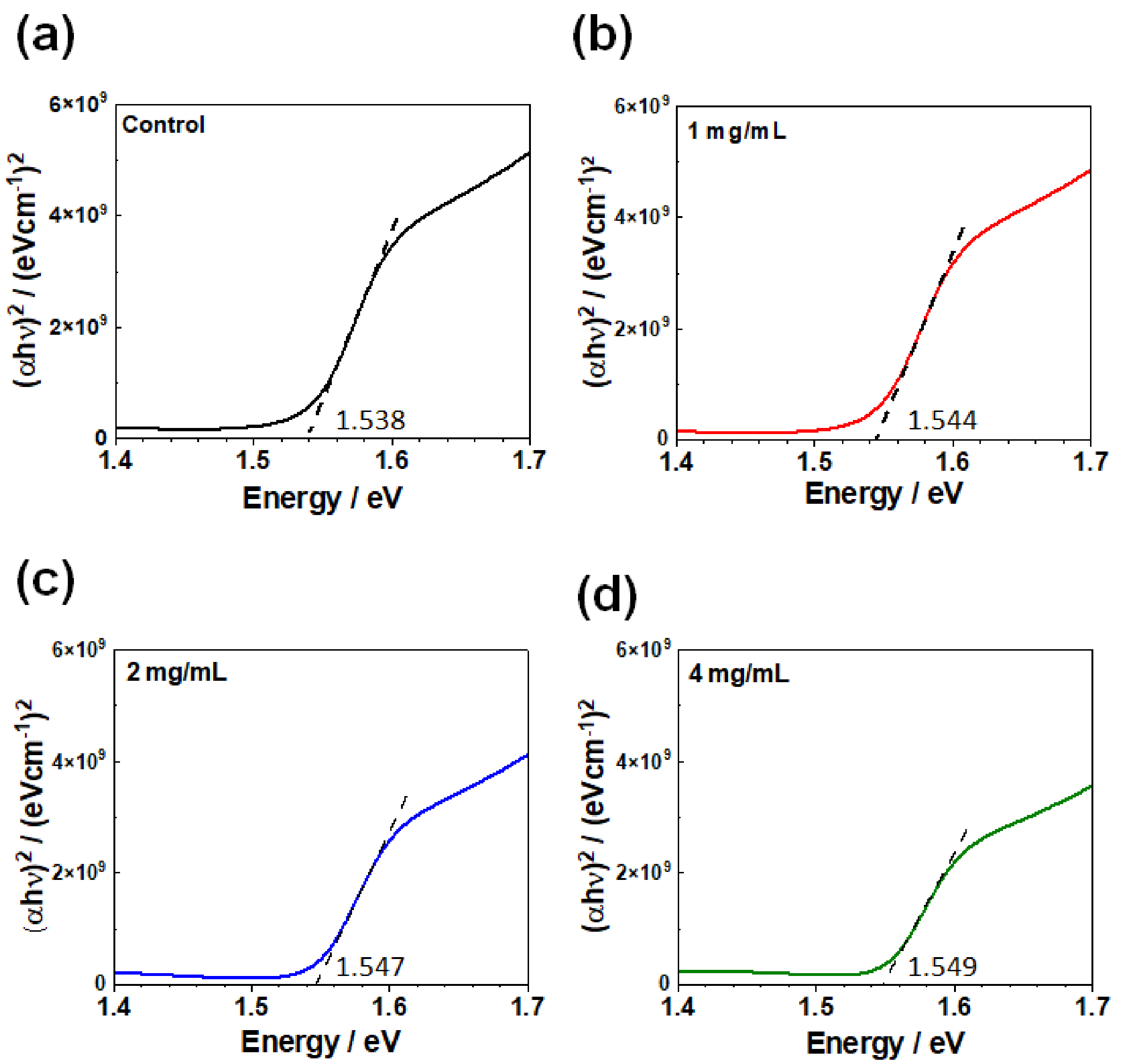

Figure S10. (a) – **(d)** Tauc plots calculated from the data shown in Figure S9. The band gaps were calculated from the intercept values of the x-axis and the values are shown.

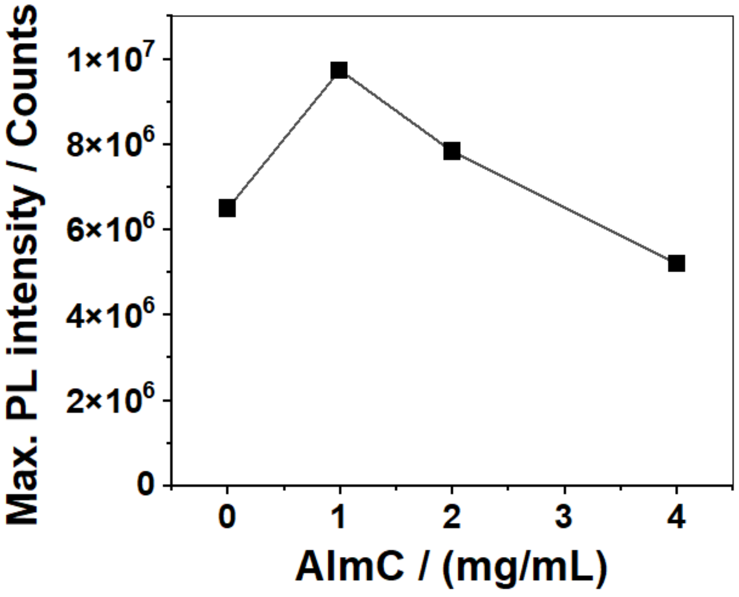

Figure S11. Maximum PL intensity of perovskite films with various concentrations of AImC. These data were obtained from Figure 4(d).

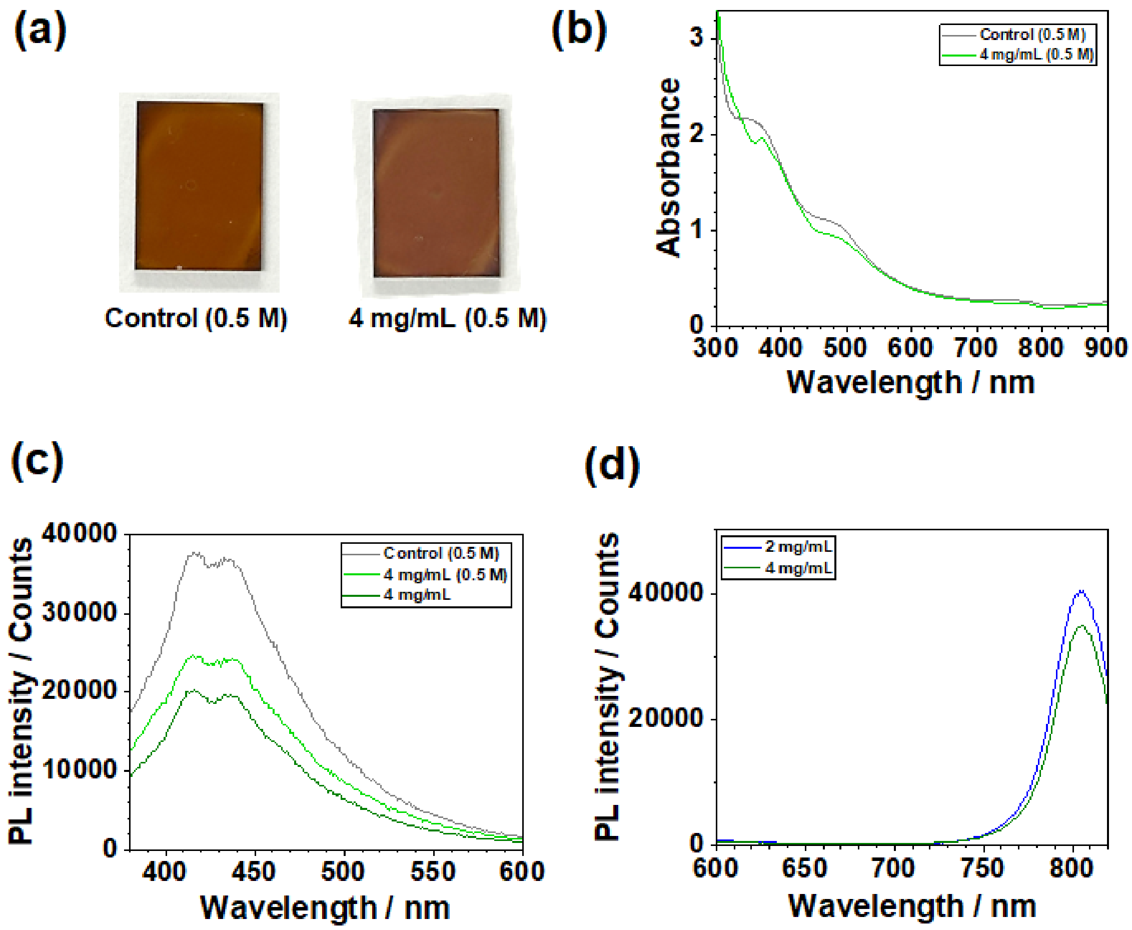

Figure S12. **(a)** Photographs of the thinner 3D perovskite prepared using 0.50 M precursor solution and subjected to 4 mg/mL AImC post-treatment. **(b)** UV-visible spectra recorded for the films from (a). **(c)** Lower wavelength PL spectra for the 4 mg/mL film as well as the control (thinner) 3D perovskite prepared using 0.50 M precursor solution and subjected to 4 mg/mL AImC treatment **(d)** Wide-wavelength scan PL spectra for the 2 mg/mL and 4 mg/mL films from Figure 4(d).

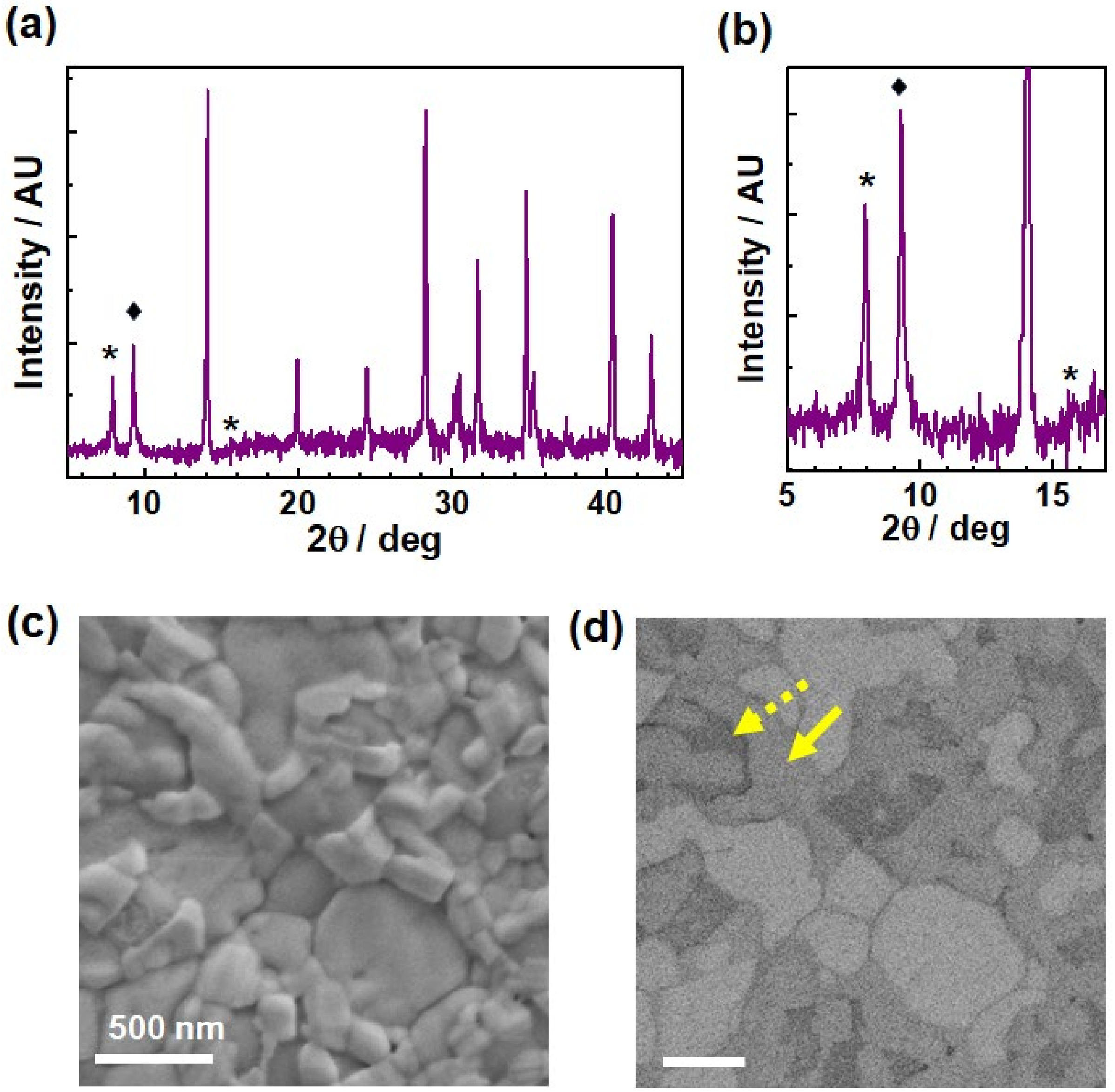

Figure S13. **(a)** XRD pattern of perovskite films treated with 7 mg/mL of AImC **(b)** Magnified XRD patterns at low angle from 5° to 17°. * indicates LD perovskite signal, diamond indicates interphase. **(c)** Top view SEM image and **(d)** BSE SEM image of the perovskite films. The full yellow arrow highlights the LD phase and the yellow dotted arrow highlights a new LD phase.

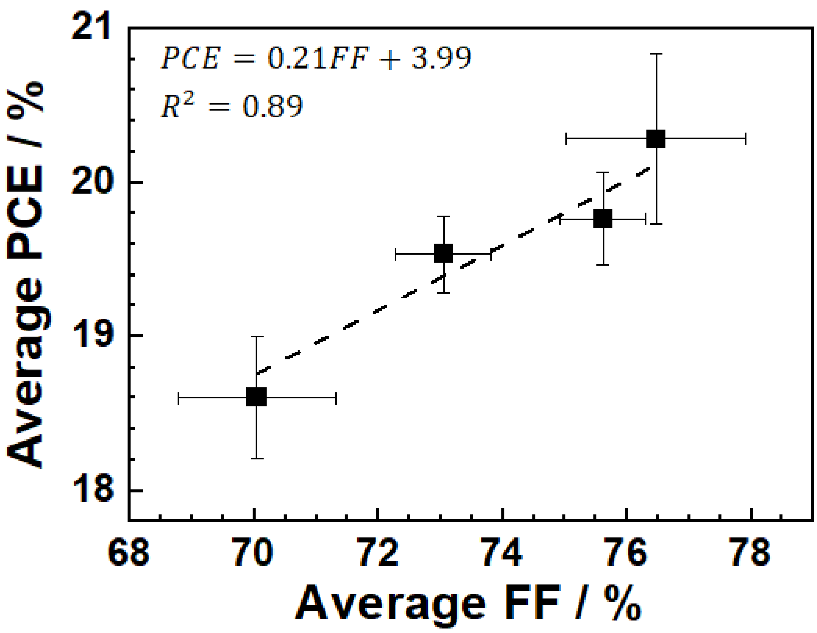

Figure S14. Average PCE vs. average FF values for the devices studied. The error bars show the respective standard deviation values.

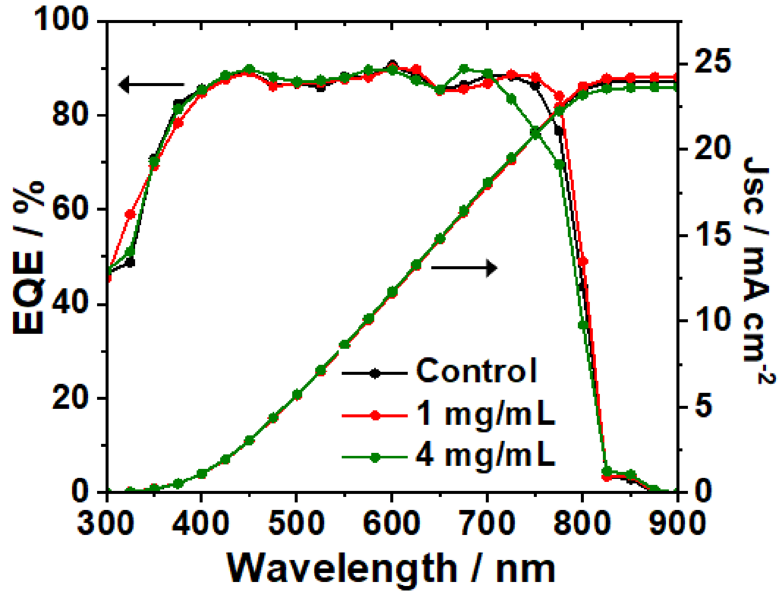

Figure S15. EQE spectra and the integrated J_{sc} of PSCs without and with AImC post-treatment.

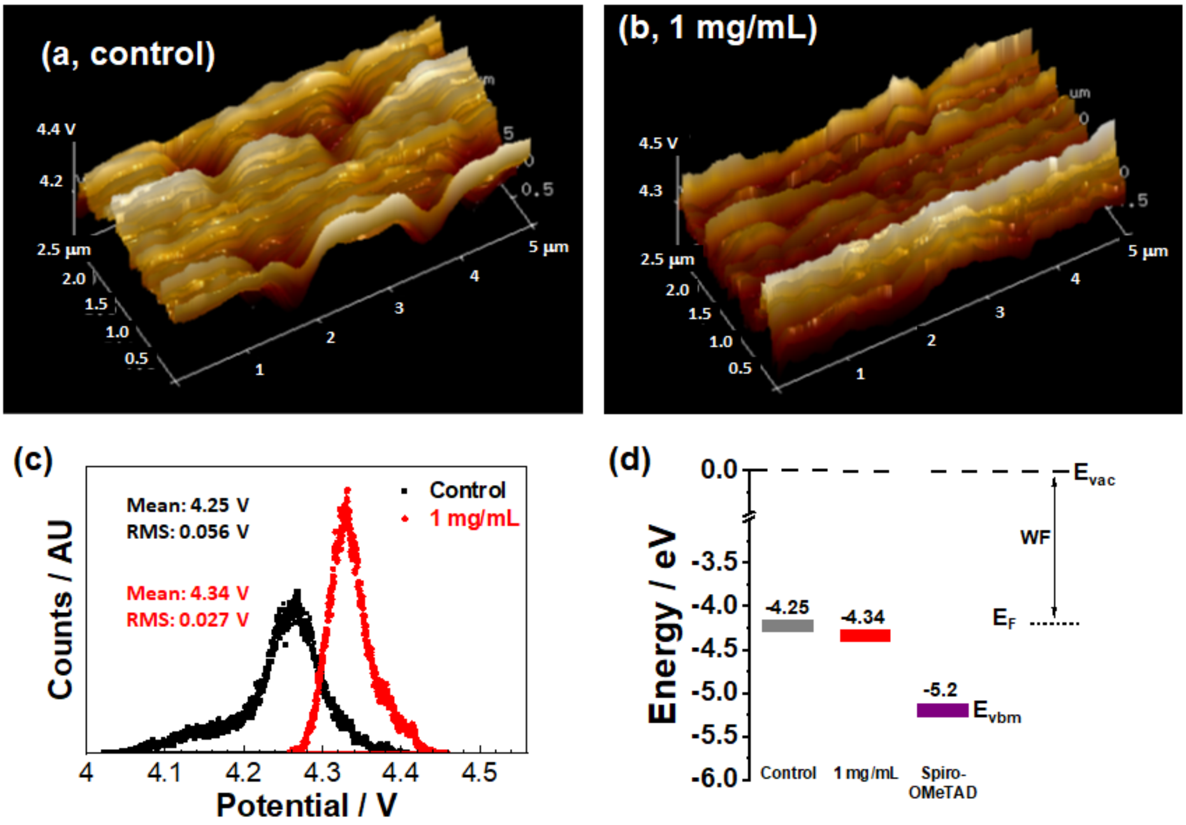

Figure S16. KPFM images of the **(a)** control and **(b)** 1 mg/mL AImC treated perovskite films. **(c)** Surface potential distributions obtained from (a) and (b). **(d)** Schematic showing the work functions (WF) and Fermi energies (E_F) for the control and 1 mg/mL AImC systems as well as the valence band maximum (E_{vbm}) for Spiro.

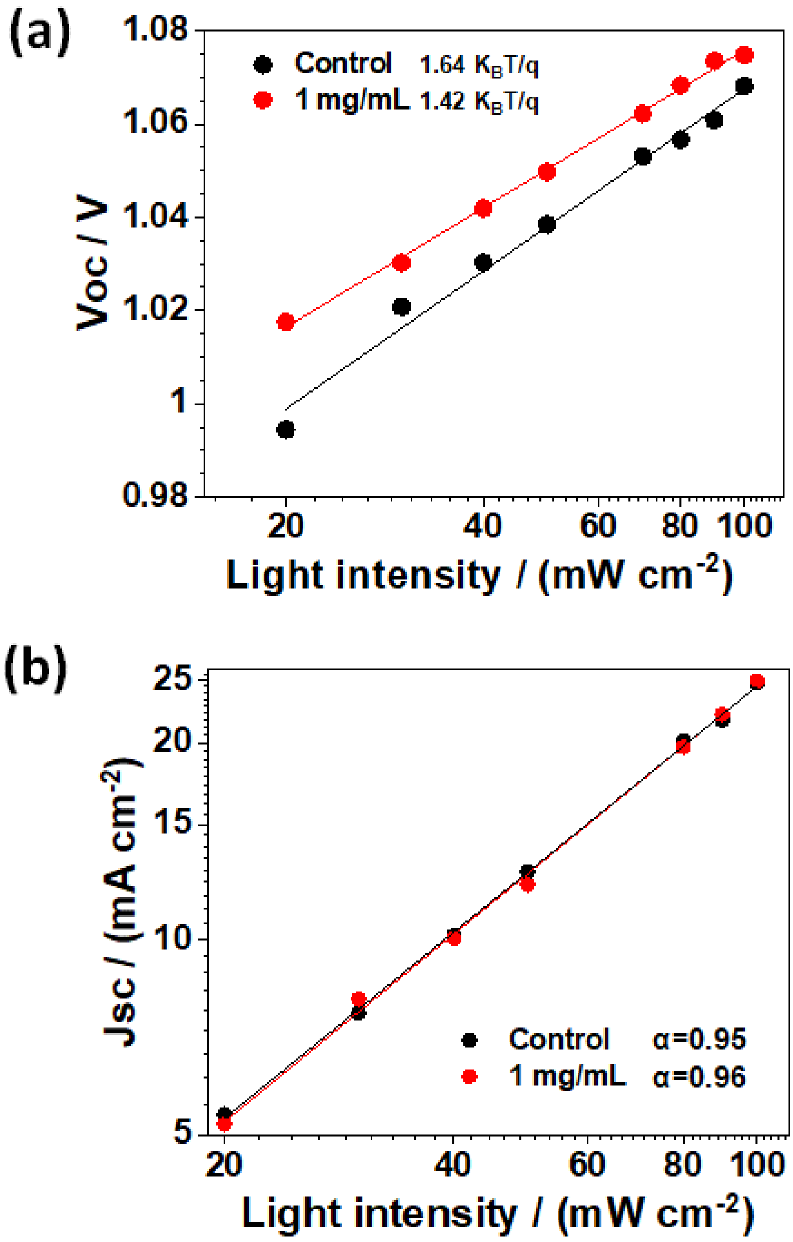

Figure S17. Light-intensity-dependence of **(a)** V_{oc} and **(b)** J_{sc} for the control and 1 mg/mL AImC treated perovskite devices.

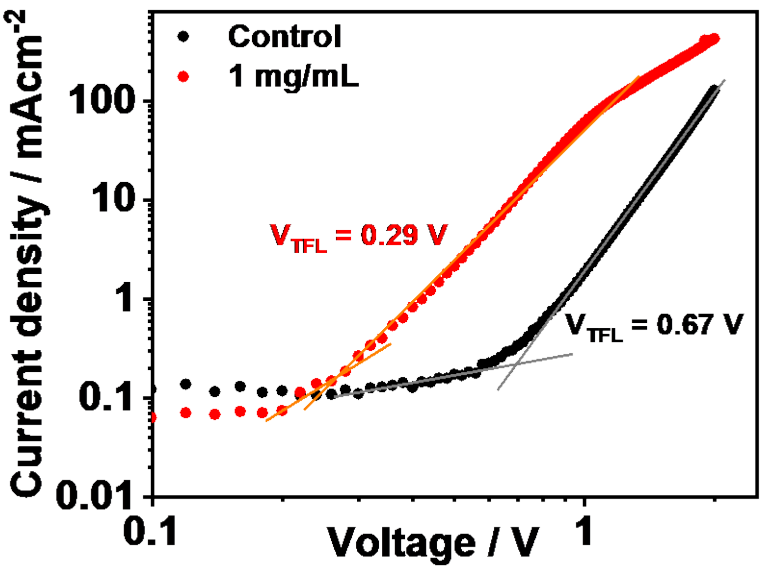

Figure S18. Space-charge limiting current measurement data for the control and AImC system obtained using glass/ITO/SnO_2/3D perovskite/LD capping layer/PCBM/Au electron-only devices.

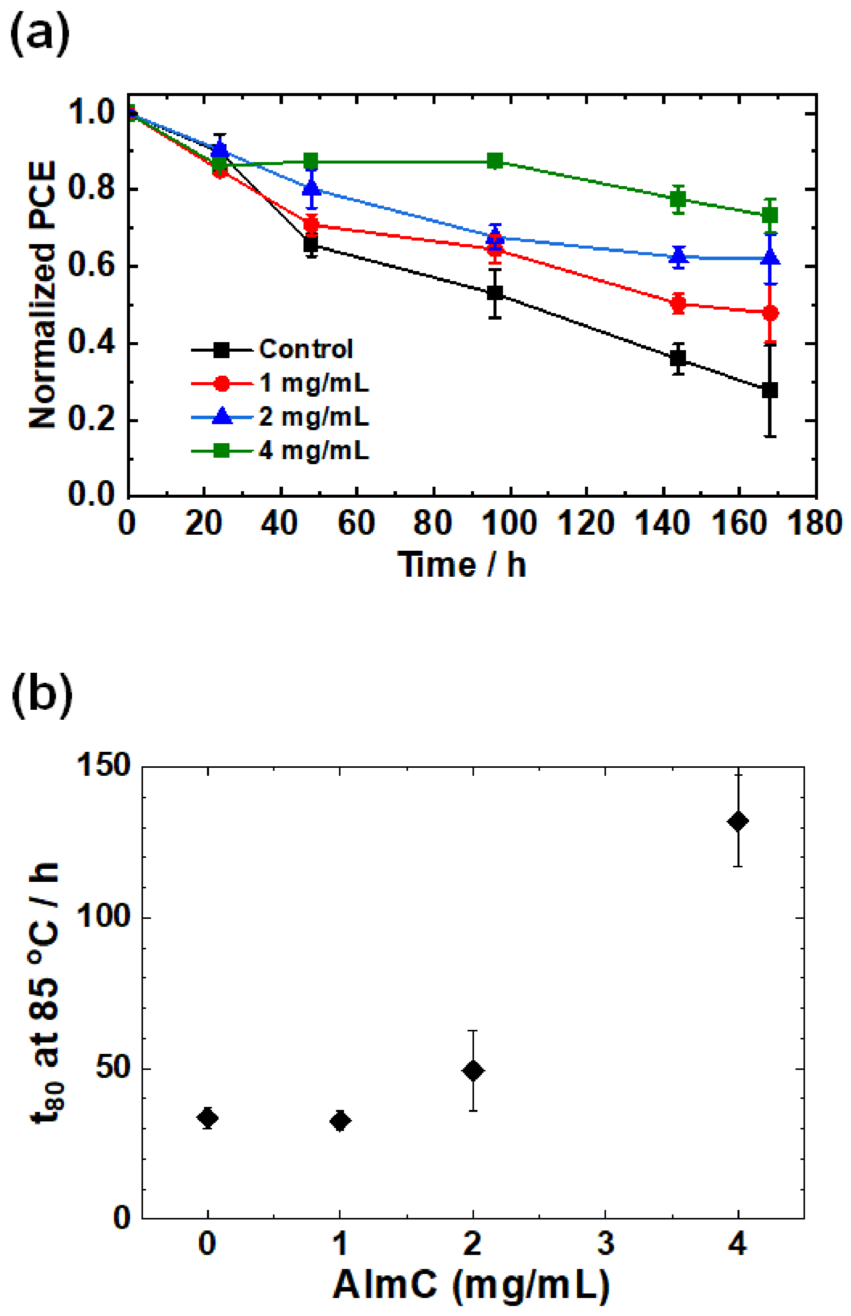

Figure S19. (a) Thermal stability of unencapsulated perovskite devices stored at 85 °C in ambient air. **(b)** Experimental t_{80} values at 85 °C using the data from (a).

Table S1. Fitting parameters from TRPL spectra of perovskite films.

Sample	τ_1 /ns	A_1 / Counts	τ_2 /ns	A_2 / Counts	τ_{ave} / ns
**Control**	62.23	107.2 (23.0%)	129.5	358.4 (77.0%)	121.08
**1 mg/mL**	79.68	80.13 (17.4%)	189.4	381.7 (82.6%)	180.46
**2 mg/mL**	41.71	56.27 (12.2%)	141.8	404.2 (87.8%)	137.87
**4 mg/mL**	40.29	34.02 (7.3%)	123.1	432.4 (92.7%)	121.01

Table S2. Photovoltaic parameters measured for the PSCs in this study.

System	Scan direction	Voc (V)	Jsc ($mA.cm^{-2}$)	FF (%)	PCE (%)	HI(%)
**Control**	Forward	1.04 ± 0.01	24.79 ± 0.40	71.03 ± 2.67	18.39 ± 0.84	6.9
	Reverse	1.05 ± 0.01	24.85 ± 0.40	75.62 ± 0.70	19.76 ± 0.30	
	**Best**	**1.06**	**25.02**	**76.2**	**20.27**	
**1 mg/mL**	Forward	1.06 ± 0.02	24.88 ± 0.47	74.40 ± 2.04	19.63 ± 0.82	3.2
	Reverse	1.07 ± 0.02	24.87 ± 0.47	76.48 ± 1.45	20.28 ± 0.55	
	**Best**	**1.06**	**25.67**	**78.87**	**21.42**	
**2 mg/mL**	Forward	1.08 ± 0.01	24.68 ± 0.30	73.00 ± 0.93	19.53 ± 0.29	0.03
	Reverse	1.08 ± 0.01	24.70 ± 0.29	73.05 ± 0.76	19.53 ± 0.25	
	**Best**	**1.09**	**25.15**	**73.21**	**20.03**	
**4 mg/mL**	Forward	1.08 ± 0.01	24.58 ± 0.29	69.73 ± 0.91	18.46 ± 0.33	0.8
	Reverse	1.08 ± 0.01	24.59 ± 0.25	70.05 ± 1.28	18.60 ± 0.40	
	**Best**	**1.08**	**24.8**	**71.85**	**19.24**	

References

1. Mao, L.; Stoumpos, C. C.; Kanatzidis, M. G., Two-Dimensional Hybrid Halide Perovskites: Principles and Promises. *J. Amer. Chem. Soc.* **2019,** *141*, 1171-1190.

2. Niu, T.; Xie, Y. M.; Xue, Q.; Xun, S.; Yao, Q.; Zhen, F.; Yan, W.; Li, H.; Brédas, J. L.; Yip, H. L., Spacer engineering of diammonium-based 2D perovskites toward efficient and stable 2D/3D heterostructure perovskite solar cells. *Adv. Energy Mater.* **2022,** *12*, 2102973.
